# Adaptations to Climate in Candidate Genes for Common Metabolic Disorders

**DOI:** 10.1371/journal.pgen.0040032

**Published:** 2008-02-15

**Authors:** Angela M Hancock, David B Witonsky, Adam S Gordon, Gidon Eshel, Jonathan K Pritchard, Graham Coop, Anna Di Rienzo

**Affiliations:** 1 Department of Human Genetics, University of Chicago, Chicago, Illinois, United States of America; 2 Department of Geophysical Sciences, University of Chicago, Illinois, United States of America; Stanford University, United States of America

## Abstract

Evolutionary pressures due to variation in climate play an important role in shaping phenotypic variation among and within species and have been shown to influence variation in phenotypes such as body shape and size among humans. Genes involved in energy metabolism are likely to be central to heat and cold tolerance. To test the hypothesis that climate shaped variation in metabolism genes in humans, we used a bioinformatics approach based on network theory to select 82 candidate genes for common metabolic disorders. We genotyped 873 tag SNPs in these genes in 54 worldwide populations (including the 52 in the Human Genome Diversity Project panel) and found correlations with climate variables using rank correlation analysis and a newly developed method termed Bayesian geographic analysis. In addition, we genotyped 210 carefully matched control SNPs to provide an empirical null distribution for spatial patterns of allele frequency due to population history alone. For nearly all climate variables, we found an excess of genic SNPs in the tail of the distributions of the test statistics compared to the control SNPs, implying that metabolic genes as a group show signals of spatially varying selection. Among our strongest signals were several SNPs (e.g., *LEPR* R109K, *FABP2* A54T) that had previously been associated with phenotypes directly related to cold tolerance. Since variation in climate may be correlated with other aspects of environmental variation, it is possible that some of the signals that we detected reflect selective pressures other than climate. Nevertheless, our results are consistent with the idea that climate has been an important selective pressure acting on candidate genes for common metabolic disorders.

## Author Summary

The human species inhabits a wide geographical range encompassing a diversity of climates, and adaptation to these climates likely played an important role in shaping genetic and phenotypic variation among populations. We hypothesized that spatially varying selective pressures related to climate shaped the frequencies of genetic variants in the energy metabolic pathway. To test this hypothesis, we examined patterns of genetic variation in 82 candidate genes for common metabolic disorders across the 52 globally dispersed populations of the Human Genome Diversity Project. We applied a combination of statistical approaches to test whether the geographic distribution of these variants could be accounted for by differing climates, consistent with a signal of spatially varying positive selection. For several climate variables, we observed signals in excess of that expected from human population history and chance alone. Significantly, many of these signals were from genes previously shown to affect cold tolerance and disease risk. Our results provide evidence that variation among human populations in susceptibility to common metabolic diseases may be due, in part, to different histories of selective pressures on genes in these disease pathways. Furthermore, our results point to additional genes and variants that are suitable targets for follow-up disease association studies.

## Introduction

The ability to cope with heat and cold stress is important for survival and reproduction of organisms in general, and especially for those exposed to extreme temperatures [1]. If phenotypes that increase heat or cold tolerance were acted upon by natural selection, the genes responsible for these phenotypes would be expected to carry detectable signatures of selection in the spatial distribution of genetic variation. Indeed, latitudinal clines of allele frequencies have been observed for several polymorphisms in humans (e.g. [2,3]) as well as in natural populations of model organisms, including Drosophila melanogaster (e.g. [4–8]), and Arabidopsis thaliana ([9–11]). Interestingly, many of the variants with latitudinal clines in *Drosophila* reside in genes involved in energy metabolism.

Adaptations to spatially varying selective pressures, such as climates, may be particularly important in human populations. Because the human species first arose in equatorial Africa, the ability to tolerate high temperatures was likely under strong long-term selection in ancestral human populations. As humans spread out of equatorial Africa to regions at higher latitudes, cold temperatures likely became important selective forces. As a consequence, traits under stabilizing selection at near-equatorial latitudes may have become deleterious at higher latitudes. In addition to thermal stress, this scenario is likely to apply to a range of selective pressures, e.g. exposure to UV radiation and to different pathogens, which may be directly influenced by and partly correlated with climate. A striking example of adaptation to an environmental variable is variation in skin pigmentation, a phenotype known to vary worldwide as a function of UV radiation [12]. Consistent with the notion that variation in skin pigmentation is adaptive, genome-wide scans for selection have detected strong signals at candidate genes for melanin production and melanocyte function [13–18]. Another possible example of spatial variation in selective pressures is sodium homeostasis. It was proposed that climate-related selection acted on the genes influencing salt and water retention and that variation in the intensity of selection partly accounts for the large inter-ethnic differences in the prevalence of salt-sensitive hypertension [19,20]. In support of this hypothesis, two recent studies reported a strong correlation between the frequencies of candidate risk variants for hypertension and latitude; this correlation was significant also when compared to that observed for a genome-wide set of random markers [21,22].

In addition to skin pigmentation and sodium homeostasis, human body shape and size also show clines with climate variables, suggesting that humans conform to the classical ecological rules that individuals inhabiting colder regions are bulkier and have shorter relative limb lengths [23,24]. More specifically, classical work by Roberts showed that mean annual temperature is significantly correlated with body mass and relative sitting height [25]. Forty years later, a re-examination of body mass measures found a strong correlation with mean annual temperature, but the slope of the correlation was smaller, probably due to nutritional changes in a subset of populations [26]. These results imply that climate had a strong influence on human morphology. Importantly, this influence can be detected even though, due to climate changes and population movements, the current climate conditions are only weak proxies for the long-term climate experienced by each population. In addition to clines of body mass measures, it was shown that basal metabolic rate is consistently elevated in indigenous circumpolar populations of North America and Siberia [27]. Although not all of this phenotypic variation has a genetic basis, these findings suggest that the metabolic state of individuals differs across populations and that this variation has adaptive significance.

The biological processes that influence tolerance to climatic extremes are likely to play important roles in the pathogenesis of common metabolic disorders, such as type 2 diabetes (T2D), obesity, dyslipidemia, and hypertension. For example, obesity risk is likely to be related to variation in energy balance, through variation in food intake, body mass index, and basal metabolic rate. Additional examples include the relationships between T2D risk and variation in blood glucose levels, between lipid levels and intake, processing and storage of lipids, and between hypertension risk and sodium retention and arterial vessel tone. Therefore, susceptibility genes for these common metabolic disorders are promising candidate targets of climate adaptations. Interestingly, these phenotypes tend to co-occur in individuals, an observation that gave rise to the definition of the metabolic syndrome as a clustering of quantitative phenotypes related to T2D, obesity, hypertension and dyslipidemia [28,29]. The clustering in individuals, along with their concomitant increase in prevalence, suggests that these diseases result from a set of shared metabolic defects and that these defects are adaptations to the life style, diet and climates experienced by human populations over long-term evolutionary time [20,30,31].

With the goal of selecting genes that experienced similar selective pressures, we used a bioinformatics approach that aims to characterize the core gene subnetwork of the metabolic syndrome phenotypes [32]. The genes in this subnetwork are likely to harbor those shared metabolic defect(s) that result in the clustering of metabolic disorders. To test the hypothesis that these genes adapted to the different climates experienced by human populations, we captured genetic variation in these genes by tag SNPs, which were genotyped in a worldwide panel of individuals [33]. Statistical tests were then performed to detect spatial patterns of allele frequencies that reflect the action of climate-related selective pressures. We find that these genes, as a group, harbor an excess of tag SNPs whose allele frequencies are correlated with climate variables. This suggests that climate was an important selective pressure acting on the biological processes underlying common metabolic disorders.

## Results

To identify spatial patterns of allele frequencies that result from adaptations to climate, we analyzed newly collected genotype data for 1083 SNPs in a worldwide panel of 1034 individuals from 54 populations, 52 of which belong to the Human Genome Diversity Project (HGDP) panel [33]. Of the 1083 SNPs, 873 are tag SNPs in 82 genes belonging to the core network of the metabolic syndrome phenotypes and 210 are control SNPs from unconstrained genomic regions. We characterized the signal of spatially varying selection in terms of the correlation between allele frequencies and four summaries of climate. To assess the significance of the correlation between genic SNP allele frequencies and climate, we compared the patterns observed at genic and control SNPs. In this analysis, the control SNPs are meant to provide an empirical null distribution for the spatial patterns of variation generated by human population history alone. Finally, to explore the performance of our approach in an empirical setting, we genotyped tag SNPs from a gene, *SLC24A5*, which is known to influence variation in skin pigmentation [13].

### Selection of Candidate Genes in the Metabolic Syndrome Core Subnetwork

In order to select a broad set of genes playing a role in metabolic disease phenotypes, we used a network-based algorithm, referred to as Molecular Triangulation [32], which uses “seed genes”, i.e. genes previously implicated in disease risk, to search through the total network of gene-gene interactions to define the disease sub-network. The seed genes can be thought of as casting a shadow on the network, the size of which is proportional to the strength of the evidence implicating the seed in the disease phenotype. Therefore, a non-seed gene interacting with a strongly supported seed gene has a high probability of belonging to the disease subnetwork; note that the disease subnetwork will include both seed and non-seed genes, all prioritized based on the network analysis.

To create the total gene network, we compiled a list of human gene-gene interactions from databases containing data from automated and human-curated literature extraction methods (BIND, preBIND, MIPS, MINT, and GeneWays) [34–38] as well as from two large-scale yeast two-hybrid studies that used human proteins [39,40]. This total interaction network contains 12,743 genes and 224,269 non-redundant gene-gene interactions.

To choose the seed genes, we conducted a large-scale literature review to identify genes that had been implicated in the etiology of T2D, obesity, hypertension, and dyslipidemia, separately, or of the metabolic syndrome as a phenotype. Each gene was scored depending on whether it was implicated in a given phenotype based on data from association studies (single and meta-analyses), animal models of the disease, pharmacological and physiological studies, or studies of the Mendelian form of the disease (seed genes for each phenotype are listed in Tables S1–S5). This information was combined to create a primary evidence score for each seed gene that summarizes the strength of the evidence implicating it in a given disease phenotype. The Molecular Triangulation algorithm uses the primary evidence scores and the total network to calculate a secondary evidence score (a function of the primary evidence scores and distances from all seed genes) for each gene and disease; the significance of the secondary evidence score is assessed by permutations [32]. The permutation-based *p*-values were combined across phenotypes for each gene and were used to rank all the genes in the total network (i.e. both seeds and non-seeds), separately. The highest ranking 39 seed and 35 non-seed genes (excluding genes that were poorly tagged in the HapMap data) were selected for analysis.

Because current knowledge of the true human gene-gene interaction network is incomplete, not all seed genes are found in the total network (e.g. *TCF7L2*) and not all strong candidate genes are identified as high ranking genes by the network analysis (e.g. *PPARG*). Therefore, we also selected an additional 8 genes with strong evidence of involvement in metabolic syndrome phenotypes. These genes are: *PPARG*, *PPARGC1A, LEPR*, *TCF7L2*, *CLOCK*, *ALMS1*, *RAPTOR* and *FRAP1*. *PPARG*, *PPARGC1A*, *LEPR* and *TCF7L2* were chosen because variation in these genes has repeatedly been found to be associated with T2D risk and/or metabolic syndrome risk [41–47]. *CLOCK* and *ALMS1* were included because they are strong functional candidates for metabolic syndrome [48–51]. *RAPTOR* was chosen because we previously observed an especially strong correlation between a microsatellite within this gene and latitude (Witonsky and Di Rienzo, unpublished data); this microsatellite had been genotyped in the HGDP at the Marshfield Clinic Center for Human Genetics [52]. In addition, the products of *RAPTOR* and *FRAP1* form a protein complex that has been implicated in energy metabolism and T2D pathways [53]. In total, we included 82 genes in the analysis (listed in Table S6).

### Testing for Spatially Varying Selection

We used the HapMap Phase II data [54] to select 873 tagging SNPs that capture most of the variation in the 82 candidate genes (see Methods). In addition, we selected 210 control SNPs. The control SNPs were chosen from non-coding regions that do not contain and are not tightly linked to evolutionarily constrained sequence elements. In order not to introduce a bias, the same ascertainment scheme was used to select genic and control SNPs (except that only one tag SNP was chosen at random from each control region) (for details, see Materials and Methods). A total of 1,083 SNPs typed in 964 individuals from the HGDP panel as well as in 37 Ewondo Beti and 33 Hausa from Cameroon yielded genotype data that passed our data quality filter (see Methods).

To check that the control SNPs were appropriately matched to the genic SNPs, we calculated the minor allele frequency and F_ST_ for each SNP in our data and compared the frequency distributions of these two statistics between the two sets of SNPs. A global F_ST_ value, based on the average squared deviation between regional allele frequencies and the global average ([55] p. 167), was calculated among the seven major geographical regions defined by Rosenberg et al. 2002 [56]. Using a two-sample Kolmogorov-Smirnov test, we found no significant difference between the distributions of genic and control SNPs for minor allele frequency (D = 0.058, *p* = 0.61) or F_ST_ (D = 0.070, *p* = 0.38). This suggests that our procedure for matching genic and control SNPs is reliable and that the control SNPs can provide an appropriate empirical null distribution for spatial patterns of variation expected for putatively neutral regions.

To test the hypothesis that climate-related selection shaped variation in the candidate genes, we obtained climate data for each population in our panel. We selected six climate variables likely to reflect the physiological effects of cold and hot climates (i.e. precipitation rate, relative humidity, minimum temperature, maximum temperature, mean temperature and short wave radiation flux). Separate values for summer and winter seasons were obtained for each of these variables. Since many of these variables are strongly correlated with one another, we reduced the dimensionality of the climate data using principal components analysis. In all analyses, we used the first two principal components (PCs) for each season, which together explained 77.6% and 78.6% of the variance in the complete data set for summer and winter variables, respectively (Tables S7 and S8). It should be noted that because of climate changes and population movements over evolutionary time, the current values of these climate variables may be only partially correlated with the long-term climatic conditions experienced by each population.

To assess the relationship between SNP allele frequency and climate, we used two methods that make different assumptions and, therefore, may be complementary. The first one, Spearman rank correlation, is a non-parametric method that does not assume a linear relationship between the variables. The second one, which will be denoted Bayesian geographic analysis, is a newly developed method that tests whether a linear relationship between allele frequency and a climate variable provides a significantly better fit than the null model alone, where the population covariance matrix provides the basis for the null model (Coop et al., in preparation). With both methods, statistical significance was assessed against the distribution of the test statistic calculated from the set of control SNPs. Although the use of control SNPs may be conservative [57], here it is necessary to reduce the number of spurious signals that would arise from the genome-wide correlation between allele frequencies and climate variables. Indeed, the control SNPs show an excess of significant correlations with all climate variables, as assessed against the theoretical null distribution (see Figure S1).

### Signals of Spatially Varying Selection on Metabolism Genes

We found that many genic SNPs had significant spatial patterns with climate variables (absolute latitude, summer PC1, summer PC2, winter PC1, winter PC2). To assess the evidence for climate adaptations in metabolism genes, we asked if genic SNPs *as a group* show an excess of signals for spatially varying selection. Our expectation was that, even if climate had exerted a strong selective pressure on these genes, only a fraction of genic SNPs would show evidence for spatially varying selection. Therefore, we asked if there was an excess of SNPs in the tail of the distribution of empirical *p*-values for the genic SNPs for several type I error (α) levels. As shown in Table 1, we found an excess of genic SNPs with *p*-values above the type I error rate for most climate variables. To rule out the possibility that this pattern was driven by linkage disequilibrium in a few genes with strong signals, we bootstrap resampled across genes and control SNPs and determined how often we found an excess (for details, see Materials and Methods). The *RAPTOR* gene had been included in our survey because of a known correlation between latitude and a microsatellite within this gene (Witonsky and Di Rienzo, unpublished data); therefore, *RAPTOR* was omitted from this analysis. (When *RAPTOR* was included in this analysis, we obtained similar results; see Table S9.) This analysis revealed strong signals for summer PC1 and winter PC1, consistent with the idea that these summaries of climate variables reflect important selective pressures that shaped the spatial distribution of allele frequencies of metabolism genes in humans. Note that these two PCs are not strongly correlated with each other as they summarize somewhat different aspects of climate (i.e. relative humidity, short-wave radiation flux and precipitation for summer PC1 and temperature variables for winter PC1) (see Table S7). Interestingly, the Bayesian geographic analysis tends to detect a stronger signal compared to the rank correlation analysis. Computer simulations showed that, under some scenarios, the Bayesian analysis is more powerful than the rank correlation analysis (G. Coop, unpublished observations); therefore, it is possible that the difference is simply due to a difference in power.

**Table 1:**
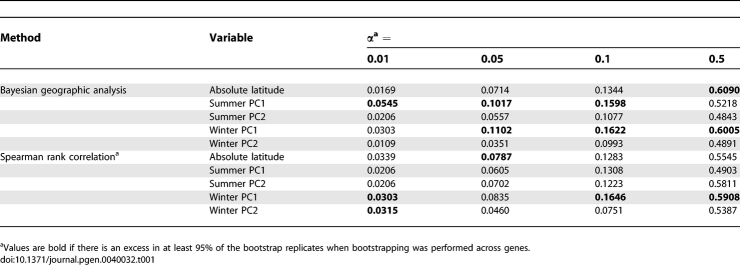
Proportion of SNPs Significant with Climate Variables Using a Parametric (Bayesian Geographic Analysis) and a Nonparametric (Spearman Rank Correlation) Method

Next, we considered the signals of spatially varying selection at individual SNPs; for this analysis, we focused on SNPs that are significant across both methods on the assumption that they represent the most robust signals. We found 72 SNPs in 32 genes that were in the top 5% of the empirical null distribution (i.e. the control SNP distribution) with the same climate variable for both methods (Table 2). At the 0.01 significance level, there were four SNPs in two genes (*FABP2* and *RAPTOR*) significant with latitude, nine SNPs in six genes (*CD36*, *DSCR1*, *MAPK14*, *PON1*, *SOD1*, and *TCF7L2*) with summer PC1, four SNPs in four genes (*CETP*, *DSCR1*, *EGFR*, and *NPPA*) significant with summer PC2, ten SNPs in five genes significant with winter PC1 (*EPHX2*, *LEPR*, *MAPK1*, *RAPTOR*, and *UCP3*) and two SNPs in two genes (*LPA* and *MMRN1*) significant with winter PC2. In total, 26 SNPs in 17 genes had empirical *p* < 0.01 with both analysis methods. Of these 17 genes, eight were seed genes, six were non-seed genes and three were other candidates.

**Table 2:**
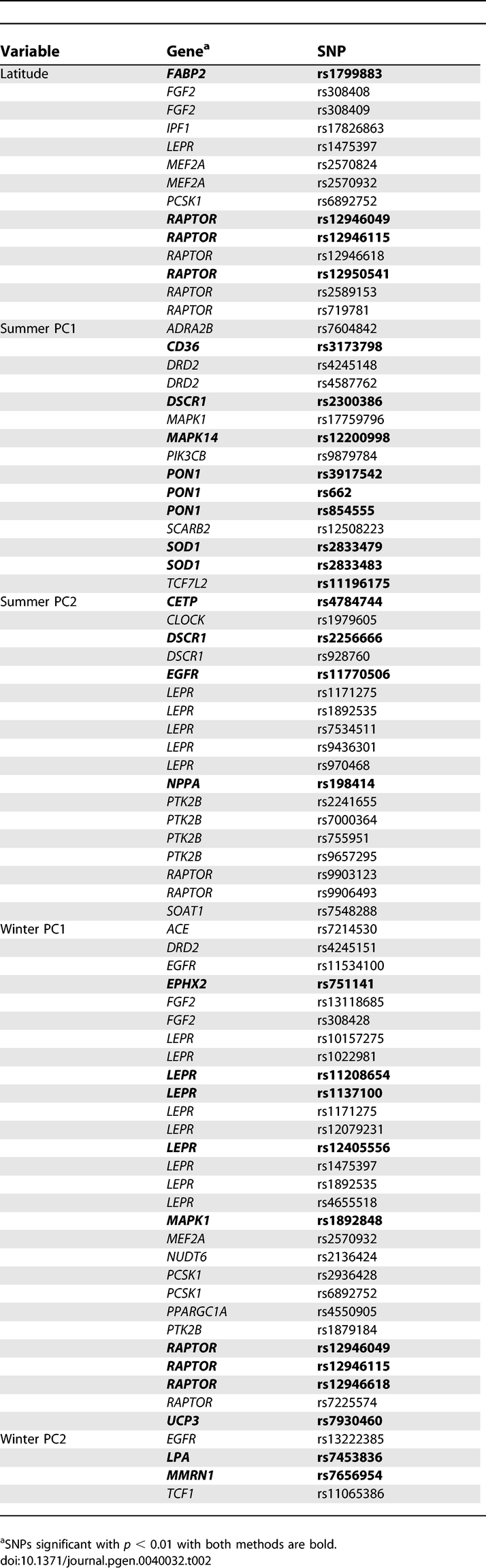
SNPs Significant with Empirical *p* < 0.05 with Both Methods

**Figure 1:**
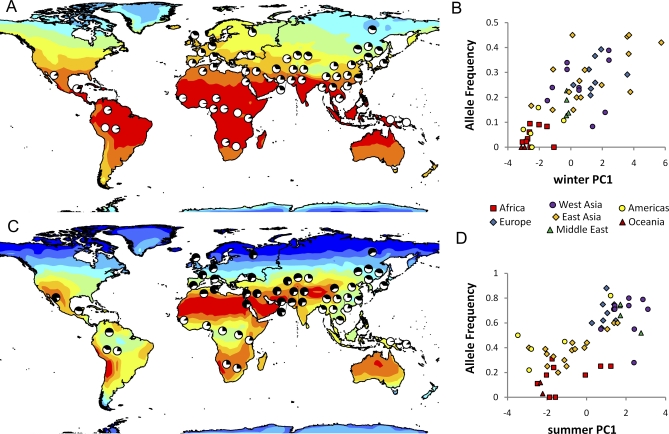
Allele Frequencies for rs12946049 in the *RAPTOR* Gene Are Mapped onto Winter Maximum Temperature (A) and Plotted against Winter PC1 (B) Allele frequencies for rs662 in the *PON1* gene are mapped onto summer SWRF (C) and plotted against summer PC1 (D).

Although we did not detect an enrichment of signals of spatially varying selection at non-synonymous SNPs, several genes carry particularly strong signals at a nonsynonymous SNP. The gene coding for the leptin receptor, *LEPR*, is among the strongest signals detected by our analysis. *LEPR* is an exceptionally strong candidate gene as it is involved in the regulation of satiety and energy balance and is part of the thermogenesis pathway [58]. A nonsynonymous SNP (K109R), rs1137100, is strongly correlated with winter PC1 (*p* < 0.01). Three SNPs in the *PON1* gene, which codes for an esterase present in high density lipoproteins (HDLs), have strong patterns with summer PC1, and one of these, rs662, is a nonsynonymous SNP (R192Q) that was shown to affect enzymatic activity *in vitro* [59] (Figure 1C and 1D). Additional nonsynonymous SNPs with strong climate signals are found in the *FABP2* and the *EPHX2* genes, which code for the intestinal fatty acid binding protein 2 and the cytoplasmic epoxide hydrolase 2, respectively. The derived allele at SNP (A54T), rs1799883, in *FABP2* increases strongly with latitude (*p* < 0.01) and SNP (R287Q), rs751141, in *EPHX2* has a significant correlation (*p* < 0.01) with winter PC1 with both methods. While these nonsynonymous SNPs are likely to have functional effects and are plausible targets of selection, we cannot rule out that selection acted on different SNPs in strong linkage disequilibrium with the non-synonymous ones.

Many genes exhibit significant climate patterns at non-coding SNPs that cannot be easily accounted for by linkage disequilibrium with nonsynonymous SNPs. Among them, the strongest signal is observed at the *RAPTOR* gene, which is involved in nutrient signaling, mitochondrial oxygen consumption and oxidative capacity [53]. Four SNPs had significant patterns with winter PC1 with *p* < 0.01 for both methods, and several additional SNPs showed significant patterns (*p* < 0.01) with either latitude or winter PC1 (*p* < 0.05) with both methods (Figure 1A and 1B). A resequencing survey did not detect any common nonsynonymous variants at this gene (Sun and Di Rienzo, unpublished observation). The *TCF7L2* gene, which shows the strongest signals in genome-wide association studies of T2D [60–64], harbors several SNPs with strong climate signals; as for *RAPTOR*, no common nonsynonymous SNPs are known at this gene [65]. SNP rs7903146 in *TCF7L2*, which is most consistently associated with T2D risk [66], is significant with summer PC1 using the Bayesian geographic analysis method (*p* = 0.019) and was suggestive using the rank correlation method (*p* = 0.057). However, another *TCF7L2* SNP, rs11196175, showed stronger patterns with all methods. This result may reflect the presence of multiple SNPs affecting *TCF7L2* function or may simply result from stochastic variation in the climate signal due to the partial correlation among alleles within a gene. Among other SNPs reported to influence T2D risk based on a large number of replication studies [67], the *PPARG* (P12A) rs1801282 is significantly correlated with latitude (*p* < 0.01 with rank correlation) while the *KCNJ11* (E23K) rs5219 is not correlated with any variable.

### The Signal of Spatially Varying Selection at Candidate SNPs for Skin Pigmentation

To better understand the expectations for genetic variation subject to spatially varying selection, we analyzed SNP allele frequencies in candidate genes for variation in skin pigmentation.

First, we used publicly available data for 6 SNPs previously genotyped in the HGDP panel [15] and subjected them to the same tests that we used to assess evidence for spatially varying selection in candidate genes for the phenotypes of the metabolic syndrome. These SNPs include the nonsynonymous SNPs in *SLC24A5* and *SLC45A2*, which were associated with normal variation in skin pigmentation [13,68], as well as a nonsynonymous SNP in *TYR* (rs1042602), which was recently associated with normal variation in hair color and with freckles [69]. The remaining variants are a nonsynonymous SNP in *OCA2* (rs1800404), a SNP in the promoter of *ASIP,* and an intronic SNP (rs1800410) in *OCA2.* These SNPs were reported to carry a signature of positive natural selection based on either haplotype structure or inter-population differentiation [13–18]. As shown in Table 3, several of these candidate SNPs showed interesting patterns with climate variables. We expected that short wave radiation flux should have the strongest signal with SNPs in skin pigmentation genes since intensity of solar radiation is likely to be the best proxy for the selective pressure acting on this phenotype [12]. Somewhat surprisingly, however, we did not see the strongest signals with short wave radiation flux. Instead, we found very strong patterns with latitude and with winter PC1. Since climate and, as a result, UV exposure at the earth surface changed over evolutionary time while latitude did not, it is possible that latitude provides a better proxy for the long-term selective pressures that shaped variation in these genes. It is also possible that the power to detect a correlation varies for different climate variables. For example, if the spatial distribution of some climate variables is similar to genome-wide patterns of population structure, neutral allele frequencies may be more strongly correlated with these climate variables, possibly leading to a greater reduction in power for some variables compared to others. Indeed, we found that the correlation between control SNP allele frequencies and climate PCs differs across variables (see Figure S1). We also found some difference between the results using the rank correlation and the Bayesian analyses. In agreement with our findings for metabolic genes, the latter tends to yield more significant signals than the nonparametric method.

**Table 3:**
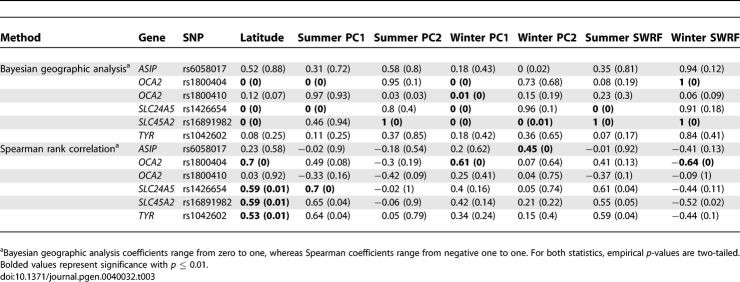
Coefficients and *p*-Values (in Parentheses) for Pigmentation Candidate SNPs.

Finally, we wanted to investigate how the signal of spatially varying selection is distributed across tag SNPs within the same gene. For this analysis, we selected the gene *SLC24A5* because of its known function in human skin pigmentation: its knockdown has a skin pigmentation phenotype in zebrafish and this phenotype can be rescued by the human *SLC24A5* [13]. In addition, the nonsynonymous polymorphism at amino acid 111 (rs1426654) was found to be associated with variation in pigmentation among African-American and African-Caribbean individuals [13]. We chose tagging SNPs for *SLC24A5* in the same way as for the metabolic syndrome candidate genes and tested them for a correlation with climate variables. The empirical *p*-values for both methods are shown in Table 4. With both methods, we found strong signals for the “causative” nonsynonymous SNP as well as for a few additional SNPs. In the case of the rank correlation analysis, some of the significant SNPs had stronger correlations with latitude, summer PC1 and summer short-wave radiation flux than the causative SNP. Similarly, with the Bayesian geographic analysis, several SNPs show a signal that is as strong as that carried by the causative SNP. These results suggest that, while there is strong signal at the *SLC24A5* gene (with as high as 60% SNPs with *p* < 0.05), there is not sufficient resolution in the SNP-based signal to distinguish the causative SNP from other correlated SNPs.

**Table 4:**
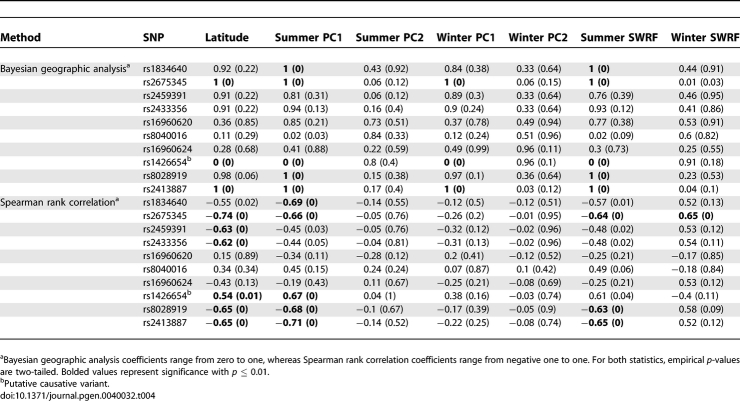
Coefficients and *p*-Values (in Parentheses) for Tag SNPs in *SLC24A5*

## Discussion

Our goal was to test the hypothesis that climate, and in particular cold and heat stress, exerted strong selective pressures on the biological processes underlying common metabolic disorders in humans. To select a group of genes likely to have experienced similar selective pressures, we exploited the knowledge-base gleaned from studies of the metabolic syndrome and its phenotypes coupled with a bioinformatics approach based on network theory. Common variation in these genes was systematically captured by genotyping tag SNPs in a worldwide population panel and the data were analyzed to characterize the spatial patterns of variation. Based on the assumption that selection varies according to measurable aspects of climate, we show that the spatial distribution of allele frequencies in genes involved in metabolic processes is significantly different from that of unconstrained SNPs. Although we cannot exclude the possibility that other environmental variables correlated with climate drove part of the signals of spatially varying selection, our results argue for a role of climate adaptations in the biological processes underlying the metabolic syndrome and its phenotypes.

The finding that variation in candidate genes for common metabolic disorders was selected because it increased tolerance to climatic extremes is supported by the link between a number of non-disease metabolic traits and the phenotypes of the metabolic syndrome. For example, variation in energy balance is likely to be related to obesity risk, through variation in food intake, body mass index, and basal metabolic rate. Basal metabolic rate has been shown to vary among species [70] as well as among human populations [26,27,71] as a function of environmental temperature, and this variation is thought to be related to the need for increased heat production to maintain homeostasis in colder climates [27]. Blood glucose levels are increased in individuals with T2D, which might increase survival in cold temperatures [72]. Elevated levels of sugar and sugar derivatives in the blood have been found in some cold-adapted species [1]. Changes in blood serum triglycerides and HDL cholesterol [73,74] with acclimation to cold environments have been documented in humans. Optimal sodium retention may differ among climates and variation in sodium retention is likely to affect hypertension risk. In hot, humid environments, high sodium retention may be necessary, whereas high sodium retention may be deleterious in cooler environments [20]. Therefore, because of the partial overlap between common metabolic disorders and cold-adaptive phenotypes, the subset of SNPs with strong signals of spatially varying selection detected in this study can be considered strong candidates for the genetic susceptibility to the metabolic syndrome.

Consistent with the idea of a link between the genetic susceptibility to metabolic disorders and adaptations to climate, many of the SNPs with strong signals in our analysis were also associated with metabolic phenotypes. As a general observation, this may not be surprising: genes were included in the analysis because of prior evidence of association with metabolic disorders. However, the specific patterns observed at SNPs in which signals of spatially varying selection coincided with evidence of association with specific phenotypes were not dictated by our study design. For example, several of the SNPs showing strong patterns with winter PC1, which summarizes mainly winter temperatures and latitude, are also known to play a role specifically in cold tolerance. Perhaps the best case is the nonsynonymous 109R allele in *LEPR*; this allele increases in frequency with decreasing winter temperatures and is associated with increased respiratory quotient (CO2 released:O2) [75], consistent with a role in non-shivering thermogenesis [75,76]. This derived allele was also reported to be associated with lower BMI, lower abdominal sagittal diameter, and lower systolic and diastolic blood pressure [75,76]. In addition, Wauters et al. [77] found an association for the *LEPR* 109R allele with fasting insulin levels and insulin response in women with impaired glucose tolerance, suggesting a role in the pathogenesis of T2D. Interestingly, a genome scan for selection signals based on the HapMap data showed that there is extended haplotype homozygosity around the *LEPR* gene, as summarized by the integrated Haplotype Score (iHS) [17]. More specifically, several SNPs in *LEPR* that had significant patterns with winter PC1 also had significant iHS values (i.e. rs1892535, rs1137100, rs1022981, and rs12405556 in Asians and rs4655518 in both Asians and CEPH Europeans). This is consistent with the idea that variants that are deleterious in hot equatorial climates become advantageous (rather than simply neutral) in colder climates.

A similarly interesting example is the signal observed at the *PON1* gene, whose protein product protects lipids from oxidation [78] and is involved in vasodilation. A derived nonsynonymous allele (192Q), which increases in frequency with latitude and affects enzymatic activity *in vitro* [59], was found to influence LDL size [79] and to be protective against coronary artery disease [80], stroke [81] and myocardial infarction risk [82]. Additional signals of spatially varying selection for nonsynonymous SNPs are found at the *FABP2* gene and the *EPHX2* gene. The FABP2 protein participates in the uptake, intracellular metabolism and transport of long-chain fatty acids. The derived allele at SNP rs1799883, which increases strongly with latitude, was found to increase affinity for long-chain fatty acids [83], consistent with a role in protection against cold temperatures by increasing BMI and increasing the fuel for heat production. In addition, it was associated with an increase in insulin resistance [83], increased plasma concentrations of cholesterol and triglycerides [84], and total abdominal and subcutaneous abdominal fat [85]. SNP (R287Q) rs751141 in *EPHX2* has a significant correlation with winter PC1 and was previously shown to modify risk of familial hypercholesterolemia. Among carriers of causative mutations for familial hypercholesterolemia, carriers of the *EPHX2* 287Q variant had higher plasma cholesterol and triglyceride levels [86]. In addition, the 287Q variant was associated with a two-fold increased risk of coronary artery calcification in African Americans, but no relationship was found in Caucasians [87].

Interestingly, for all four nonsynonymous SNPs discussed above, the derived allele increases in frequency outside Africa. However, two of these derived alleles (*LEPR* 109R and *PON1* 192Q) decrease risk to disease phenotypes (obesity and CAD), while the other two (*FABP2* 54T and *EPHX2* 287Q) increase risk for disease traits (obesity and coronary artery calcification). Moreover, the frequencies of two additional derived alleles that protect against T2D (i.e. rs7903146 in *TCF7L2* and P12A (rs1805192) in *PPARG*) and show significant climate signals also increase outside Africa. Therefore, these derived alleles have opposing effects on disease susceptibility, even though they are all likely to be involved in adaptations to cold climates. This observation contrasts with the results of a survey of variants influencing risk to hypertension; in all cases, the frequencies of the protective alleles were found to increase outside Africa, but some of these alleles were ancestral rather than derived [22]. A possible explanation is that the impact of climate adaptations on the susceptibility to common diseases varies depending on the biological process underlying the specific disease trait. For example, it is reasonable to expect that adaptations to heat and cold stress involve a different set of genes and biological processes, e.g. sodium homeostasis and energy metabolism, respectively. Therefore, the evidence for climate adaptations in candidate genes for metabolic disorders does not lead to the expectation that these conditions will systematically occur at higher frequency in one ethnic group compared to another. On the other hand, one might speculate that climate adaptations involving different biological processes account in part for the distribution of metabolic profiles and disease-related traits across ethnic groups [88].

The idea that alleles that confer adaptations to climate have opposing effects on disease susceptibility is not entirely unexpected; this is probably because positive selection acted on phenotypes that are related, but distinct from the metabolic disorders that reach high prevalence among contemporary populations or that account only for a portion of all patients. For example, a haplotype in the *TCF7L2* gene was reported to carry a signature of positive selection and this haplotype includes the derived allele at SNP rs7903146 that protects against T2D [66]. Interestingly, this haplotype is associated with higher body mass index and altered concentrations of the hunger-satiety hormones ghrelin and leptin, suggesting that it conferred a selective advantage on energy metabolism. Our finding that SNP rs7903146 has a significant signal of spatially varying selection and that the derived allele increases in frequency outside Africa is consistent with this proposal. Furthermore, it illustrates how an adaptation to climate may partially, but not entirely overlap with the disease phenotype.

A key feature of our approach was the use of a network algorithm to choose a set of candidate genes likely to have experienced similar selective pressures, hence leading to the expectation that these genes, *as a group*, would show a signal of spatially varying selection. A network approach seemed particularly appropriate because of the widely accepted idea that the clustering of quantitative phenotypes typical of the metabolic syndrome is due to a small set of shared metabolic defects. By choosing seed genes implicated in all or most of the phenotypes that characterize the metabolic syndrome, we aimed to extract information regarding the core portion of the metabolic syndrome subnetwork; the genes in this core subnetwork are likely to be responsible for the concomitant increase in prevalence of metabolic syndrome phenotypes and to have been targets of natural selection in ancestral populations. The observed excess of signals of spatially varying selection in this group of genes suggests that our strategy for identifying candidate genes with similar evolutionary histories was successful. Interestingly, we found a similar proportion of seed and non-seed genes with signals of spatially varying selection, consistent with the idea that network theory provides a useful framework for elucidating the impact of natural selection.

From the population genetics standpoint, our results suggest that searching for a correlation between allele frequencies and specified environmental variables complements other approaches to detecting the signature of selection that also compare allele frequencies across populations. For example, there is a long history of using F_ST_ values for detecting adaptations to local environments [89–91] and this approach has been recently applied to genome-wide variation data [92,93]. Even though spatially varying selection may lead to high F_ST_ values, a strong and significant correlation between allele frequencies and environmental variables can be detected even if allele frequencies at opposite extremes of the environmental range are not highly differentiated. This is what we observed, for example, in the *RAPTOR* gene, which carries the strongest signal with latitude. Six SNPs are significant with empirical *p* < 0.05; however, when compared to the HapMap data, the F_ST_ values for these same SNPs are unremarkable, with 12% to 86% of HapMap SNPs showing more extreme values.

The power and accuracy of our approach is likely to depend on a number of factors, including the strength of selection relative to gene flow. If selection is not sufficiently strong to spread favored alleles between subdivided populations, allele frequencies may not show the expected clines with the appropriate environmental variable. This may be the case for some of the signals observed in this study. For example, the allele frequencies at candidate SNPs for skin pigmentation are not most strongly correlated with the variable, i.e. short-wave radiation, that most closely matches the selective pressure acting on these variants. Indeed, several of these variants reach high frequency only in Eurasian populations living west of the Himalayas, while East Asian populations at similar latitudes have allele frequencies as low as those found in Sub-Saharan Africa [15]. Although further work is necessary to estimate the power of our approach for a range of selective coefficients and migration rates, our empirical investigation based on the skin pigmentation SNPs suggests that strong signals can be detected even if the spatial distribution of allele frequencies does not match exactly the spatial distribution of the intensity of selection. Additional factors that may affect the power and accuracy of our approach include threshold effects on fitness, so that a correlation between an allele frequency and an environmental variable is expected only over a portion of the range of environmental variation. Furthermore, as noted for skin pigmentation variants [15], parallel mutations that arose in different populations may obscure the expected correlation with the relevant environmental variable.

Although we tested for a correlation between allele frequencies and climate variables, it is unclear whether all the signals of spatially varying selection reported here are the result of adaptations to climate rather than other environmental variables. To the extent that other features of the environment are partially correlated with climate, our method is unlikely to distinguish between selective pressures. For example, UV radiation, which was included among the climate variables in our analysis, is correlated with the other climate variables and with latitude (data not shown). Clearly, UV radiation influences environmental temperature and, therefore, it is expected to exert a selective pressure on genes involved in diverse biological processes, from energy metabolism to melanin production. In some cases, climate may be an important determinant of other aspects of the environment. An important example is the diversity of parasitic and infectious disease species, which is known to decrease with latitude mainly as a result of climatic factors [94]. Likewise, resource availability, which in turn may influence subsistence and diet, is likely to be partially correlated with climate (see, for example [95,96]). Therefore, the signals of spatially varying selection, detected in this study are likely to be the result of both the direct and indirect influence of climate on human genetic variation; they may also be due to selective pressures entirely unrelated to climate, but partially correlated with it. Despite these caveats, our results indicate that searching for spatial patterns of allele frequencies that correlate with environmental variables will add to our knowledge concerning the history of selective pressures acting on the human genome.

## Materials and Methods

### Population samples.

We genotyped 971 individuals from 52 worldwide populations from the CEPH Human Genome Diversity Project (HGDP) panel [33] and 10 duplicate controls. From the original panel of 1064 samples, 93 were excluded from analysis because they had been inferred to be either from members of first-degree relative pairs, duplicates or atypical [97]. In all analyses, we combined all individuals from South African Bantu populations into one group, which we considered to be different from the Bantu sample from Kenya. In addition, we genotyped 72 individuals from two Cameroonian populations. We included 38 individuals from the Ewondo Beti, who belong to the Bantu language group, and 34 individuals from the Chadic-speaking Hausa; all Cameroonian individuals were sampled in Yaounde. This study was approved by the University of Chicago Institutional Review Board.

### Candidate gene selection using network analysis.

Primary evidence scores used for running the molecular triangulation program were assigned based on the types of evidence found in the literature review. For each type of evidence, points were added to the primary evidence score in the following manner: ten points were added if there was significant evidence of association from a meta-analysis, one point was added if there was significant evidence of association in a single analysis, four points were added if a Mendelian phenotype results from mutations in a given gene, four points were added if the gene product is a drug target, one point was added if the gene was found to affect a given phenotype in an animal model, one point was added if there was physiological evidence a gene was involved in a phenotype and for genes with at least one of the previous types of evidence, we gave one point for genes that fell under a linkage peak. In order to be included as a “seed” gene in the network analysis, a gene needed to have a primary evidence score ≥3, except in the case of metabolic syndrome, where we only required a score ≥1. The score required was lower for metabolic syndrome because this phenotype is likely to represent a defect in the core metabolic pathway and because few studies have examined metabolic syndrome directly. We chose genes using the molecular triangulation method in two ways: (1) by running the algorithm on each phenotype separately and then combining the *p*-values and (2) by running the algorithm on genes with strength of evidence scores reflecting evidence for all phenotypes together. For the separate phenotype analysis, we first ran the molecular triangulation algorithm on each phenotype separately and summed the *p*-values associated with the secondary evidence scores across all phenotypes. Then, we chose 50 “seed” and 50 “non-seed” genes with the lowest sums of the *p*-values. For the combined phenotype analysis, we summed the strength of evidence scores for all phenotypes, ran the molecular triangulation algorithm, and chose the most significant 20 “seed” and 20 “non-seed” genes. This list of genes was reduced by choosing the 39 seed and 35 non-seed genes that had the best coverage of tag SNPs in the HapMap (a total of 34 out 140 genes were omitted due to poor tagging in the HapMap). We also selected an additional 8 genes with strong evidence of involvement in MetS phenotypes that were not present in the network. These genes are described in greater detail in the Results section. In total, we included 82 genes in the analysis.

### Selection of SNPs in candidate genes.

Genic SNPs were selected from among HapMap Phase II (Build 35) SNPs located in a region that spans from 100 kb upstream to 100 kb downstream of each of the 82 genes. The frequency of the global minor allele (GMA) for each SNP was estimated based on the allele counts of the combined sample of 180 unrelated HapMap individuals (60 CEU parents, 60 YRI parents, 30 JPT + 30 CHB chosen at random from the 90 Asian samples). Given these allele frequencies, the SNPs selected for genotyping were further filtered using the following steps. First, SNPs with GMA frequency less than 10% or greater than 90% in all three regional populations were omitted. Second, SNPs from step 1 were sorted into tag bins using ldSelect (v 1.0) [98], applying an r^2^ threshold of 0.8. The process of sorting into tag bins was done both for the global sample and the non-African (CEU + ASN) sample. Third, tag SNPs were selected from each bin observed in the global sample; in addition, tag SNPs were selected from bins that were found only in the non-African sample. If a tag bin had multiple tag SNPs, the SNP with the highest Illumina assay score was chosen. SNPs were also preferentially included if they were nonsynonymous or if they had been previously associated with disease phenotypes. For all genes, tag SNPs were chosen from among those bins whose span (the smallest region containing all SNPs in the bin) intersected the region between 5 kb upstream and 5 kb downstream of the gene.

### Selection of control SNPs.

To control for the effect of population structure in assessing the relationship between climate variables and allele frequencies at candidate genes, we genotyped a set of control SNPs from unconstrained genomic regions. These regions were chosen from among noncoding regions that are neither conserved nor in strong LD with deeply conserved sequence elements. To select unconserved regions, sequence conservation was calculated over sliding windows (100 bp window size, 1 bp step size) in the human-chicken, human-zebrafish, human-mouse and human-rat genome alignments, which were obtained from the UCSC Genome Browser (http://genome.ucsc.edu), and a region was considered conserved if sequence identity exceeded 70%. Then, using estimates of the population recombination rate parameter ρ (=4Nr, where N is the effective population size and r is the cross-over rate between adjacent sites) [99], regions were selected to be at least 200 kb in length and at least 40 ρ units away from regions conserved between human and chicken or between human and zebrafish, to be unconserved between human and mouse or between human and rat, and not to contain coding regions. Among these regions, we selected a subset so that their lengths matched the distribution of lengths for genic regions. SNPs from these regions were chosen using a procedure similar to the procedure used in the selection of genic SNPs. Briefly, tag SNPs were chosen so that their allele frequency distribution matched that of SNPs in genic regions. One SNP was selected at random from the set of tag SNPs each control region.

### SNP genotyping.

1130 SNPs were genotyped using the Illumina BeadArray technology at the UCLA Southern California Genotyping Consortium Facility. Thirty-nine SNPs had 100% missing data. In addition, data were filtered so that three SNPs with missing data for more than 5% of the genotypes, three SNPs with high population-specific missing data, and two monomorphic SNPs were excluded from analyses. Seven HGDP individuals, one Hausa and one Beti individuals with more than 10% missing data were excluded from analyses. Data quality was assessed by genotyping 10 duplicate samples; we found only one genotyped pair with a discrepant call, for an error rate of 9.25 × 10^−5^.

Tag SNPs in the *SLC24A5* gene were genotyped using TaqMan Assays on Demand, for which 2μl of 40ng/μL DNA was added to 3.5 μL TaqMan SNP Genotyping MasterMix, 0.125 Assay on Demand solution and 2.5 μL ddH_2_O. Fluorescence was visualized on an Applied Biosystems 7900 HT Fast Real Time PCR machine. Data quality was assessed by genotyping 7 duplicate samples; we did not find any discrepant call in these duplicates.

For all analyses, ancestral state was inferred based on the human-chimpanzee genome alignment. When the ancestral state could not be unambiguously inferred, the major allele in the combined African HGDP populations was assumed to be ancestral.

### Climate variables.

Climate data were obtained for each population based on coordinates provided with the HGDP panel. For the Cameroonian samples, coordinates for the birthplaces of each individual's grandparents were obtained from Mapquest (http://www.mapquest.com). We selected climate variables separately for the summer and winter seasons from the NCEP/NCAR database [100]; they included: precipitation rate, relative humidity, minimum temperature, maximum temperature, mean temperature and short wave radiation flux. Values for summer and winter seasons were obtained for each variable by averaging variables over these seasons. We ran principal components analysis separately for summer and winter on a matrix that included the six climate variables as well as latitude and longitude using the prcomps() function in R [101]. Latitude and longitude were included because they might capture the long-term climate of human populations better than the climate variables measured over the past 50 years. Principal components analysis results in new variables, which are linear combinations of the original data weighted by the eigenvectors. Eigenvectors for these PCs and the proportion of the variance explained by each PC are shown in Tables S7 and S8.

### Detecting SNPs with climate patterns.

We used two different methods to determine whether SNP allele frequencies at the candidate genes had been shaped by selective pressures related to climate. In the first method, a Spearman rank correlation coefficient is calculated for each SNP and each climate variable or PC. The data from the control SNPs are used to provide an empirical null distribution of rank correlation coefficients that reflects the history of human migrations and population structure. Specifically, for the rank correlation coefficient value of each genic SNP we found the percentile value in the control SNP distribution; this percentile value is referred to as the empirical p value for a given genic SNP and a given climate variable or PC. The Spearman rank correlation method does not assume that the relationship between SNP allele frequency and climate variable or PC is linear.

The second method, which we refer to as Bayesian geographic analysis, is designed to test for a correlation between an environmental variable and the frequency of an allele across populations, while accounting for differences in sample size and the neutral covariation of allele frequencies among populations. This approach involves two stages. First, we use a set of SNPs (which can be treated as randomly chosen for our purposes and which were typed by Conrad et al., 2006 [102]) to estimate the covariance matrix (Ω) whose entries give the covariance of allele frequencies between pairs of populations. To estimate this matrix we make a number of assumptions. The allele counts at a SNP in a population are assumed to be a binomial draw from the unknown underlying population allele frequency. We assume the population allele frequency within a population marginally has a normal distribution where the mass of the normal distribution below zero is placed as a point mass on zero and the mass above one is placed on one. The population frequency across populations is assumed to have a multivariate normal distribution, with an unknown covariance matrix Ω and mean μ. Given these assumptions we used Markov Chain Monte Carlo (MCMC) to sample the posterior distribution of the covariance matrix (using standard priors). Second, for each genic SNP and each climate variable, we tested for correlation with the climate variable (whose values across the 52 populations is a vector Y), while controlling for population structure using the covariance matrix estimated in the first step. To perform this test we included a linear effect βY (where β is the coefficient) in the mean of the multivariate normal, and estimated the posterior of β using MCMC. To summarize the posterior in a single test score we recorded the proportion of the posterior density, which was above zero. If the data has no strong signal of an effect of the environmental variable (Y) the posterior of β will be reasonably symmetrically distributed around zero resulting in scores close to 0.5, a posterior strongly skewed towards positive β will have a score close to one, while a posterior distribution strong skewed towards negative β will give a score close to zero. We also ran a test for each environmental variable on each control SNP and use these scores to estimate, for each environmental variable, an empirical two-tailed *p*-value for each genic SNP.

We included the two Cameroonian populations (Hausa and Beti) when we ran the Spearman method, but we excluded these populations when we ran the Bayesian geographic analysis method since the covariance matrix was created from a dataset that did not include these populations.

### Testing for excess significance for climate variables.

Since we performed many tests and the SNPs within a given gene are not independent, we asked whether the genic SNPs as a group show a larger proportion of low p values than expected by chance. To this end, we chose several type I error levels (α = 0.01, 0.05, 0.1 and 0.5) and, for each variable, we calculated the proportion of genic SNPs with empirical *p*-values less than α. Furthermore, because the SNPs within any given genic region are not completely independent while the control SNPs are, it is possible that the observed excess of genic SNPs with *p* < α is driven by a large number of correlated SNPs within one or few genes rather than by SNPs in many genes. To distinguish between these two possibilities, we bootstrap re-sampled across genic and control SNPs and recalculated, for each of 1000 bootstrap replicates, the proportion of SNPs with *p*-values lower than each cutoff. This distribution of proportions was then used to get a measure of the confidence that the excess we observed was robust to the choice of genes and control SNPs. More specifically, for each replicate, we drew a set of 81 genes with replacement from the original set of genes and a set of 210 controls with replacement from the list of control SNPs. Then, we calculated empirical *p*-values for each of the genic SNPs using the distribution of control SNPs. Next, we determined the proportion of genic SNPs that had *p*-values less than or equal to each α cutoff. Finally, we found the proportion of bootstrap replicates where the proportion of SNPs with *p*-values less than or equal to the cutoff was greater than expected by chance.

## Supporting Information

Figure S1 Correlation between Climate Variables and Control SNPs (A) shows the frequency distribution of the Spearman rank correlation test statistic (ρ) for the control SNPs for each of the climate variables. The critical values for α = 0.01 from the theoretical distribution are shown by vertical lines. Panel B shows the distribution of Spearman rank correlation *p*-values from the theoretical distribution. The horizontal line shows the expectation if there was no effect due to population structure (i.e., if the *p*-values were uniformly distributed).
(2.0 MB TIF)

Table S1 Literature Review Results for Hypertension (636 KB DOC)

Table S2 Literature Review Results for Type 2 Diabetes (513 KB DOC)

Table S3 Literature Review Results for Obesity (1.0 MB DOC)

Table S4 Literature Review Results for Dyslipidemia (374 KB DOC)

Table S5 Literature Review Results for Metabolic Syndrome (113 KB DOC)

Table S6 Selection of Candidate Genes for Genotyping (93 KB DOC)

Table S7 Eigenvectors from Principal Components Analysis (35 KB DOC)

Table S8 Proportion of Variance Explained by Each Principal Component (30 KB DOC)

Table S9 Proportion of SNPs Significant with *RAPTOR* Included in the Analysis (37 KB DOC)

## Abbreviations

HGDP: Human Genome Diversity Project
T2D: type 2 diabetes
