# Supplementary material for: Adaptations to Climate in Candidate Genes for Common Metabolic Disorders
Source: PLoS Genet. 2008 Feb 15;4(2):e32. doi: 10.1371/journal.pgen.0040032 (PMC2242814; doi:10.1371/journal.pgen.0040032)
Supplement: Supplementary file 1 [file pgen.0040032.st001.doc]

**Supplementary Table 1. Literature review results for hypertension**

| Gene | Gene ID | Mendelian phenotype | Animal model | Common phenotype (associations) | | Linkage* | Physiology | Drug target | Differentially expressed genes in SHR rats compared to normotensive rats |
| --- | --- | --- | --- | --- | --- | --- | --- | --- | --- |
|  |  |  |  | Single analysis | Meta analysis |  |  |  |  |
| ACE | 1636 |  |  | [1] |  |  | [2] | [3] |  |
| ACPL2 | 92370 |  |  |  |  |  |  |  | [4] |
| ADD1 | 118 |  |  | [1] |  | [5] | [6] |  |  |
| ADD2 | 119 |  |  | [7] |  |  | [6] |  |  |
| ADRA1B | 147 |  |  |  |  |  | [6] |  | [4] |
| ADRA2B | 151 |  |  | [1] |  |  | [6] |  |  |
| ADRB1 | 153 |  |  | [8] |  |  | [6] | [8] |  |
| ADRB2 | 154 |  |  | [9] & [1] |  |  | [6] |  |  |
| ADRB3 | 155 |  |  | [9] & [1] |  |  | [6] |  |  |
| AFM | 173 |  |  |  |  |  |  |  | [10] |
| AGT | 183 |  | [11] | [1] | [12] | [1] | [2] |  |  |
| AGTR1 | 185 |  | [13] | [14] |  |  | [2] | [14] |  |
| AGTR2 | 186 |  |  | [6] |  |  | [6] |  |  |
| TRAK2 | 66008 |  |  |  |  |  |  |  | [4] |
| AMPD2 | 271 |  |  |  |  |  |  |  | [4] |
| AOX1 | 316 |  |  |  |  |  |  |  | [4] |
| APOB | 338 |  |  | [9] |  |  |  |  |  |
| APOC3 | 345 |  |  | [9] |  |  |  |  |  |
| AR | 367 |  |  | [9] |  |  |  |  |  |
| ARHGAP8 | 23779 |  |  |  |  |  |  |  | [4] |
| BDKRB1 | 623 |  |  |  |  |  | [15] |  |  |
| BDKRB2 | 624 |  |  |  |  |  | [15] |  |  |
| BHMT | 635 |  |  |  |  |  |  |  | [10] |
| BZRP | 706 |  |  |  |  |  |  |  | [4] |
| CD36 | 948 |  | [16] |  |  |  |  |  |  |
| CLCNKB | 1188 | [17] |  |  |  |  |  |  |  |
| COPS5 | 10987 |  |  |  |  |  |  |  | [4] |
| CORIN | 10699 |  |  | [18] |  |  | [19] |  |  |
| CXCL16 | 58191 |  |  |  |  |  |  |  | [4] |
| CYBA | 1535 |  |  |  |  |  | [20] |  |  |
| CYP2C8 | 1558 |  |  |  |  |  | [15] |  |  |
| CYP2D7P1 | 1564 |  |  |  |  |  |  |  | [4] |
| CYP3A5 | 1577 |  |  | [21] |  |  |  |  |  |
| CYP11B1 | 1584 | [22] |  |  |  |  |  |  |  |
| CYP11B2 | 1585 | [23] |  | [1] |  | [1] | [2] |  |  |
| CYP17A1 | 1586 | [22] |  |  |  |  |  |  |  |
| CYP21A2 | 1589 | [17] |  |  |  |  |  |  |  |
| DCPS | 28960 |  |  |  |  |  |  |  | [4] |
| DRD2 | 1813 |  |  | [9] & [1] |  |  |  |  |  |
| DDT | 1652 |  |  |  |  |  |  |  | [10] |
| DGAT2 | 84649 |  |  |  |  |  |  |  | [4] |
| DPEP1 | 1800 |  |  |  |  |  |  |  | [10] |
| DPT | 1805 |  |  |  |  |  |  |  | [4] |
| DRD1 | 1812 |  |  | [24] |  |  |  |  |  |
| ECE1 | 1889 |  | [25] | [26] |  |  | [27] |  |  |
| EDN1 | 1906 |  | [28]; [29] | [1] |  |  | [6] |  |  |
| EDN2 | 1907 |  |  |  |  |  | [6] |  |  |
| EDNRA | 1909 |  |  |  |  |  | [6] |  |  |
| EDNRB | 1910 |  |  |  |  |  | [15] |  |  |
| ELA1 | 1990 |  |  |  |  |  |  |  | [10] |
| EMILIN1 | 11117 |  | **[30]** |  |  |  |  |  |  |
| EPHX2 | 2053 |  |  | [31] |  |  |  |  | [10] |
| ESR1 | 2099 |  |  |  |  |  | [20] |  |  |
| ESR2 | 2100 |  |  |  |  |  | [20] |  |  |
| F2 | 2147 |  |  |  |  |  | [15] |  |  |
| F2R | 2149 |  |  |  |  |  | [15] |  |  |
| FHL2 | 2274 |  |  |  |  |  |  |  | [4] |
| FMO3 | 2328 |  |  |  |  |  |  |  | [4] |
| FUT3 | 2525 |  |  | [32] |  |  |  |  |  |
| FUT4 | 2526 |  |  |  |  |  |  |  | [4] |
| GCK | 2645 |  |  | [24] |  |  |  |  |  |
| GM2A | 2760 |  |  |  |  |  |  |  | [4] |
| GNAS | 2778 |  |  | [24] |  |  | [6] |  |  |
| GNB3 | 2784 |  |  | [1] |  |  | [6] |  |  |
| GNB5 | 10681 |  |  |  |  |  |  |  | [4] |
| GPX1 | 2876 |  |  |  |  |  | [20] |  |  |
| GRK4 | 2868 |  | [33] |  |  |  |  |  |  |
| GSTM1 | 2944 |  |  |  |  |  | [20] |  | [4] |
| GSTO | 9446 |  |  |  |  |  |  |  | [10] |
| GSTT2 | 2953 |  |  |  |  |  |  |  | [10] |
| GULP1 | 51454 |  |  |  |  |  |  |  | [4] |
| GYS1 | 2997 |  |  | [34] |  |  |  |  |  |
| HMOX2 | 3163 |  |  |  |  |  |  |  | [10] |
| HSD11B1 | 3290 | [35] | [36] | [37] |  |  | [20] |  |  |
| HSD11B2 | 3291 | [22] |  | [1] |  |  | [20] |  |  |
| HYOU1 | 10525 |  |  |  |  |  |  |  | [4] |
| IL18 | 3606 |  |  |  |  |  |  |  | [4] |
| INSR | 3643 |  |  | [9] & [1] |  |  |  |  |  |
| KCNJ1 | 3758 | [17] |  |  |  |  |  |  |  |
| KIF1C | 10749 |  |  |  |  |  |  |  | [4] |
| KLK1 | 3816 |  | [38] |  |  |  | [20] |  |  |
| LANCL1 | 10314 |  |  |  |  |  |  |  | [4] |
| LGALS1 | 3956 |  |  |  |  |  |  |  | [10] |
| LMNA | 4000 |  |  | [9] |  |  |  |  |  |
| LPL | 4023 |  |  | [1] |  |  |  |  |  |
| LY6H | 4062 |  |  |  |  |  |  |  | [4] |
| MAMDC2 | 256691 |  |  |  |  |  |  |  | [4] |
| MAT2A | 4144 |  |  |  |  |  |  |  | [10] |
| MCAM | 4162 |  |  |  |  |  |  |  | [4] |
| MEP1B | 4225 |  |  |  |  |  |  |  | [10] |
| MFAP3 | 4238 |  |  |  |  |  |  |  | [4] |
| MGLL | 11343 |  |  |  |  |  |  |  | [10] |
| MTHFR | 4524 |  |  | [24] |  |  |  |  |  |
| MYH9 | 4627 |  |  |  |  |  |  |  | [4] |
| NCOA3 | 8202 |  |  |  |  |  |  |  | [4] |
| NDUFC2 | 4718 |  |  |  |  |  |  |  | [4] |
| NOS1 | 4842 |  |  |  |  |  | [15] |  |  |
| NOS2A | 4843 |  |  | [1] |  | [39] | [27] |  |  |
| NOS3 | 4846 |  | [40] | [1] |  | [41] | [6] |  |  |
| NPHS1 | 4868 |  |  |  |  |  | [20] |  |  |
| NPHS2 | 7827 |  |  |  |  |  | [20] |  |  |
| NPPA | 4878 |  |  | [24] |  |  | [6] |  |  |
| NPR1 | 4881 |  |  | [1] |  |  |  |  |  |
| NPR2 | 4882 |  |  | [1] |  |  |  |  |  |
| NQO1 | 1728 |  |  | [24] |  |  |  |  |  |
| NR3C2 | 4306 | [22] | [42] |  |  |  |  |  |  |
| PCSK6 | 5046 |  |  |  |  |  |  |  | [4] |
| PIGL | 9487 |  |  |  |  |  |  |  | [4] |
| PKD1 | 5310 | [15] |  |  |  |  |  |  |  |
| PKD2 | 5311 | [15] |  |  |  |  |  |  |  |
| PLOD2 | 5352 |  |  |  |  |  |  |  | [4] |
| PMVK | 10654 |  |  |  |  |  |  |  | [4] |
| PRKCQ | 5588 |  |  |  |  |  |  |  | [4] |
| PTGIS | 5740 |  |  |  |  |  | [43] |  |  |
| PTGES | 9536 |  |  |  |  |  | [44] |  |  |
| PTGS2 | 5743 |  |  |  |  |  | [44] |  |  |
| PTPN1 | 5770 |  |  | [9] |  |  |  |  |  |
| PTPRJ | 5795 |  |  |  |  |  |  |  | [10] |
| REN | 5972 |  |  | [1] |  | [1] | [6] |  |  |
| RENBP | 5973 |  |  |  |  |  | [6] |  |  |
| ALDH1A2 | 8854 |  |  |  |  |  |  |  | [10] |
| RPL30 | 6156 |  |  |  |  |  |  |  | [10] |
| RPL34P1 | 26514 |  |  |  |  |  |  |  | [4] |
| RPP25 | 54913 |  |  |  |  |  |  |  | [4] |
| ACSM3 | 6296 |  |  | [1] |  |  |  |  | [10] |
| SCNN1A | 6337 | [45] |  | [46] |  |  | [6] |  |  |
| SCNN1B | 6338 | [22] | [47] | [24] |  |  | [6] |  |  |
| SCNN1G | 6340 | [34] | [48] |  |  |  |  |  |  |
| SDF2 | 6388 |  |  |  |  |  |  |  | [4] |
| SF3B1 | 23451 |  |  |  |  |  |  |  | [4] |
| SGK | 6446 |  |  | [49] |  |  | [50] |  |  |
| SHBG | 6462 |  |  |  |  |  |  |  | [4] |
| SLC2A4 | 6517 |  |  |  |  |  |  |  | [4] |
| SLC12A1 | 6557 | [51] |  | [20] |  |  | [52] |  |  |
| SLC12A3 | 6559 | [53] |  |  |  |  |  |  |  |
| SLC17A1 | 6568 |  |  |  |  |  |  |  | [10] |
| SMO | 6608 |  |  |  |  |  |  |  | [4] |
| SOD1 | 6647 |  |  |  |  |  | [15] |  | [10] |
| SORL1 | 6653 |  |  |  |  |  |  |  | [4] |
| SPARC | 6678 |  |  |  |  |  |  |  | [10] |
| SQRDL | 58472 |  |  |  |  |  |  |  | [4] |
| CRSP3 | 9439 |  |  | [54] |  |  |  |  |  |
| TBXA2R | 6915 |  |  |  |  |  | [15] |  |  |
| TBXAS1 | 6916 |  |  |  |  |  | [15] |  |  |
| TGFB1 | 7040 |  |  | [24] |  |  | **[30]** |  |  |
| TH | 7054 |  |  | [24] |  |  |  |  |  |
| THUMPD1 | 55623 |  |  |  |  |  |  |  | [4] |
| TIMM17A | 10440 |  |  |  |  |  |  |  | [10] |
| TMOD2 | 29767 |  |  |  |  |  |  |  | [4] |
| TNFRSF1B | 7133 |  |  | [55] |  | [55] |  |  |  |
| TNFSF12 | 8742 |  |  |  |  |  |  |  | [4] |
| TBXAS1 | 6916 |  |  |  |  |  | [15] |  |  |
| UBE2D2 | 7322 |  |  |  |  |  |  |  | [4] |
| UGT1A6 | 54578 |  |  |  |  |  |  |  | [10] |
| VDR | 7421 |  |  | [9] |  |  |  |  |  |
| VEGF | 7422 |  |  |  |  |  | [56] |  |  |
| WNK1 | 65125 | [57] | [58] |  |  |  |  |  |  |
| WNK3 | 65267 |  | [59] |  |  |  |  |  |  |
| WNK4 | 65266 | [57] |  |  |  | [1] |  |  |  |
| WRB | 7485 |  |  |  |  |  |  |  | [4] |

* Evidence was recorded only for genes that lie under linkage peaks and are also implicated by other types of evidence.

References:

1.	Agarwal, A., G.H. Williams, and N.D.L. Fisher, *Genetics of human hypertension.* Trends in Endocrinology and Metabolism, 2005. **16**(3): p. 127-133.

2.	Yagil, Y. and C. Yagil, *The search for the genetic basis of hypertension.* Curr Opin Nephrol Hypertens, 2005. **14**(2): p. 141-7.

3.	Swales, J.D., *The renin-angiotensin system as a target for therapeutic intervention.* J Cardiovasc Pharmacol, 1994. **24 Suppl 2**: p. S1-5.

4.	Hubner, N., et al., *Integrated transcriptional profiling and linkage analysis for identification of genes underlying disease.* Nat Genet, 2005. **37**(3): p. 243-53.

5.	Cusi, D., et al., *Polymorphisms of alpha-adducin and salt sensitivity in patients with essential hypertension.* Lancet, 1997. **349**(9062): p. 1353-7.

6.	Marteau, J.B., et al., *Genetic determinants of blood pressure regulation.* J Hypertens, 2005. **23**(12): p. 2127-43.

7.	Wang, J.G., et al., *Association between hypertension and variation in the alpha- and beta-adducin genes in a white population.* Kidney Int, 2002. **62**(6): p. 2152-9.

8.	Parikh, H. and L. Groop, *Candidate Genes for Type 2 Diabetes.* Reviews in Endocrine & Metabolic Disorders, 2004. **5**(2): p. 151-176.

9.	Bouchard, C., et al., *Obesity Gene Map Database*. 2006.

10.	Hinojos, C.A., et al., *Combined genealogical, mapping, and expression approaches to identify spontaneously hypertensive rat hypertension candidate genes.* Hypertension, 2005. **45**(4): p. 698-704.

11.	Tanimoto, K., et al., *Angiotensinogen-deficient mice with hypotension.* J Biol Chem, 1994. **269**(50): p. 31334-7.

12.	Sethi, A.A., B.G. Nordestgaard, and A. Tybjaerg-Hansen, *Angiotensinogen gene polymorphism, plasma angiotensinogen, and risk of hypertension and ischemic heart disease: a meta-analysis.* Arterioscler Thromb Vasc Biol, 2003. **23**(7): p. 1269-75.

13.	Ito, M., et al., *Regulation of blood pressure by the type 1A angiotensin II receptor gene.* Proc Natl Acad Sci U S A, 1995. **92**(8): p. 3521-5.

14.	Bonnardeaux, A., et al., *Angiotensin II type 1 receptor gene polymorphisms in human essential hypertension.* Hypertension, 1994. **24**(1): p. 63-9.

15.	Oparil, S. and M.A. Weber, *Hypertension*. 2nd ed. 2005.

16.	Pravenec, M., et al., *Genetics of Cd36 and the clustering of multiple cardiovascular risk factors in spontaneous hypertension.* J Clin Invest, 1999. **103**(12): p. 1651-7.

17.	Lifton, R.P., A.G. Gharavi, and D.S. Geller, *Molecular mechanisms of human hypertension.* Cell, 2001. **104**(4): p. 545-56.

18.	Dries, D.L., et al., *Corin gene minor allele defined by 2 missense mutations is common in blacks and associated with high blood pressure and hypertension.* Circulation, 2005. **112**(16): p. 2403-10.

19.	Yan, W., et al., *Corin, a transmembrane cardiac serine protease, acts as a pro-atrial natriuretic peptide-converting enzyme.* Proc Natl Acad Sci U S A, 2000. **97**(15): p. 8525-9.

20.	Iwai, N., et al., *Genetic analysis of 22 candidate genes for hypertension in the Japanese population.* J Hypertens, 2004. **22**(6): p. 1119-26.

21.	Givens, R.C., et al., *CYP3A5 genotype predicts renal CYP3A activity and blood pressure in healthy adults.* J Appl Physiol, 2003. **95**(3): p. 1297-300.

22.	New, M.I., et al., *Monogenic low renin hypertension.* Trends Endocrinol Metab, 2005. **16**(3): p. 92-7.

23.	Lusis, A.J., R. Mar, and P. Pajukanta, *Genetics of atherosclerosis.* Annu Rev Genomics Hum Genet, 2004. **5**: p. 189-218.

24.	Hirschhorn, J.N., et al., *A comprehensive review of genetic association studies.* Genet Med, 2002. **4**(2): p. 45-61.

25.	Yanagisawa, H., et al., *Role of Endothelin-1/Endothelin-A receptor-mediated signaling pathway in the aortic arch patterning in mice.* J Clin Invest, 1998. **102**(1): p. 22-33.

26.	Funke-Kaiser, H., et al., *Differential binding of transcription factor E2F-2 to the endothelin-converting enzyme-1b promoter affects blood pressure regulation.* Hum Mol Genet, 2003. **12**(4): p. 423-33.

27.	Lowenstein, C.J., et al., *Cloned and expressed macrophage nitric oxide synthase contrasts with the brain enzyme.* Proc Natl Acad Sci U S A, 1992. **89**(15): p. 6711-5.

28.	Kurihara, Y., et al., *Elevated blood pressure and craniofacial abnormalities in mice deficient in endothelin-1.* Nature, 1994. **368**(6473): p. 703-10.

29.	Kurihara, Y., et al., *Aortic arch malformations and ventricular septal defect in mice deficient in endothelin-1.* J Clin Invest, 1995. **96**(1): p. 293-300.

30.	Zacchigna, L., et al., *Emilin1 links TGF-beta maturation to blood pressure homeostasis.* Cell, 2006. **124**(5): p. 929-42.

31.	Fornage, M., et al., *Polymorphism of the soluble epoxide hydrolase is associated with coronary artery calcification in African-American subjects: The Coronary Artery Risk Development in Young Adults (CARDIA) study.* Circulation, 2004. **109**(3): p. 335-9.

32.	Cakir, B., et al., *Association of the Lewis genotype with cardiovascular risk factors and subclinical carotid atherosclerosis: the Atherosclerosis Risk in Communities (ARIC) study.* J Intern Med, 2004. **255**(1): p. 40-51.

33.	Felder, R.A., et al., *G protein-coupled receptor kinase 4 gene variants in human essential hypertension.* Proc Natl Acad Sci U S A, 2002. **99**(6): p. 3872-7.

34.	Schalin-Jantti, C., et al., *Polymorphism of the glycogen synthase gene in hypertensive and normotensive subjects.* Hypertension, 1996. **27**(1): p. 67-71.

35.	Jamieson, A., et al., *Apparent cortisone reductase deficiency: a functional defect in 11beta-hydroxysteroid dehydrogenase type 1.* J Clin Endocrinol Metab, 1999. **84**(10): p. 3570-4.

36.	Paterson, J.M., et al., *Metabolic syndrome without obesity: Hepatic overexpression of 11beta-hydroxysteroid dehydrogenase type 1 in transgenic mice.* Proc Natl Acad Sci U S A, 2004. **101**(18): p. 7088-93.

37.	Lovati, E., et al., *Molecular basis of human salt sensitivity: the role of the 11beta-hydroxysteroid dehydrogenase type 2.* J Clin Endocrinol Metab, 1999. **84**(10): p. 3745-9.

38.	Meneton, P., et al., *Cardiovascular abnormalities with normal blood pressure in tissue kallikrein-deficient mice.* Proc Natl Acad Sci U S A, 2001. **98**(5): p. 2634-9.

39.	Rutherford, S., et al., *Chromosome 17 and the inducible nitric oxide synthase gene in human essential hypertension.* Hum Genet, 2001. **109**(4): p. 408-15.

40.	Huang, P.L., et al., *Hypertension in mice lacking the gene for endothelial nitric oxide synthase.* Nature, 1995. **377**(6546): p. 239-42.

41.	Arngrimsson, R., et al., *Evidence for a familial pregnancy-induced hypertension locus in the eNOS-gene region.* Am J Hum Genet, 1997. **61**(2): p. 354-62.

42.	Berger, S., et al., *Mineralocorticoid receptor knockout mice: pathophysiology of Na+ metabolism.* Proc Natl Acad Sci U S A, 1998. **95**(16): p. 9424-9.

43.	Nakayama, T., et al., *Splicing mutation of the prostacyclin synthase gene in a family associated with hypertension.* Biochem Biophys Res Commun, 2002. **297**(5): p. 1135-9.

44.	Swan, S.K., et al., *Effect of cyclooxygenase-2 inhibition on renal function in elderly persons receiving a low-salt diet. A randomized, controlled trial.* Ann Intern Med, 2000. **133**(1): p. 1-9.

45.	Schaedel, C., et al., *Lung symptoms in pseudohypoaldosteronism type 1 are associated with deficiency of the alpha-subunit of the epithelial sodium channel.* J Pediatr, 1999. **135**(6): p. 739-45.

46.	Iwai, N., et al., *Association of a sodium channel alpha subunit promoter variant with blood pressure.* J Am Soc Nephrol, 2002. **13**(1): p. 80-5.

47.	Pradervand, S., et al., *A mouse model for Liddle's syndrome.* J Am Soc Nephrol, 1999. **10**(12): p. 2527-33.

48.	Volk, K.A., et al., *Overexpression of the epithelial Na+ channel gamma subunit in collecting duct cells: interactions of Liddle's mutations and steroids on expression and function.* J Biol Chem, 2005. **280**(18): p. 18348-54.

49.	von Wowern, F., et al., *Genetic variance of SGK-1 is associated with blood pressure, blood pressure change over time and strength of the insulin-diastolic blood pressure relationship.* Kidney Int, 2005. **68**(5): p. 2164-72.

50.	Xu, B.E., et al., *WNK1 activates SGK1 to regulate the epithelial sodium channel.* Proc Natl Acad Sci U S A, 2005. **102**(29): p. 10315-20.

51.	Simon, D.B., et al., *Bartter's syndrome, hypokalaemic alkalosis with hypercalciuria, is caused by mutations in the Na-K-2Cl cotransporter NKCC2.* Nat Genet, 1996. **13**(2): p. 183-8.

52.	Quaggin, S.E., et al., *Localization of the renal Na-K-Cl cotransporter gene (Slc12a1) on mouse chromosome 2.* Mamm Genome, 1995. **6**(8): p. 557-8.

53.	Simon, D.B., et al., *Gitelman's variant of Bartter's syndrome, inherited hypokalaemic alkalosis, is caused by mutations in the thiazide-sensitive Na-Cl cotransporter.* Nat Genet, 1996. **12**(1): p. 24-30.

54.	Sato, N., et al., *A haplotype in the human Sur2 gene is associated with essential hypertension.* J Hum Hypertens, 2006. **20**(1): p. 87-90.

55.	Glenn, C.L., et al., *Linkage and association of tumor necrosis factor receptor 2 locus with hypertension, hypercholesterolemia and plasma shed receptor.* Hum Mol Genet, 2000. **9**(13): p. 1943-9.

56.	Hayman, R., et al., *Preeclampsia: the endothelium, circulating factor(s) and vascular endothelial growth factor.* J Soc Gynecol Investig, 1999. **6**(1): p. 3-10.

57.	Wilson, F.H., et al., *Human hypertension caused by mutations in WNK kinases.* Science, 2001. **293**(5532): p. 1107-12.

58.	Zambrowicz, B.P., et al., *Wnk1 kinase deficiency lowers blood pressure in mice: a gene-trap screen to identify potential targets for therapeutic intervention.* Proc Natl Acad Sci U S A, 2003. **100**(24): p. 14109-14.

59.	Rinehart, J., et al., *WNK3 kinase is a positive regulator of NKCC2 and NCC, renal cation-Cl- cotransporters required for normal blood pressure homeostasis.* Proc Natl Acad Sci U S A, 2005. **102**(46): p. 16777-82.
