# Supplementary material for: Adaptations to Climate in Candidate Genes for Common Metabolic Disorders
Source: PLoS Genet. 2008 Feb 15;4(2):e32. doi: 10.1371/journal.pgen.0040032 (PMC2242814; doi:10.1371/journal.pgen.0040032)
Supplement: Supplementary file 2 [file pgen.0040032.st002.doc]

Supplementary Table 2. Literature review results for type 2 diabetes.

| Gene | Gene ID | Mendelian phenotype | Animal model | Common phenotype (associations) | | Linkage* | Physiology | Drug target |
| --- | --- | --- | --- | --- | --- | --- | --- | --- |
|  |  |  |  | Single analysis | Meta analysis |  |  |  |
| **ABCA1** | 19 |  |  | [1] |  |  |  |  |
| **ABCC8** | 6833 |  |  | [1-3] | [4] |  | [5] | [6] |
| **ABCC9** | 10060 |  |  | [3] |  |  |  |  |
| **ACACA** | 31 |  |  |  |  |  | [7] |  |
| **ADIPOQ** | 9370 |  | [8] | [9] |  |  | [5] |  |
| **ACE** | 1636 |  |  | [2] |  |  |  |  |
| **ADIPOR1** | 51094 |  |  |  |  |  | [10] |  |
| **ADIPOR2** | 79602 |  |  |  |  |  | [10] |  |
| **ADRB2** | 154 |  |  | [11], [2] | [12] |  |  |  |
| **ADRB3** | 155 |  |  | [11] |  |  |  |  |
| **AGPAT2** | 10555 | [9] |  |  |  |  |  |  |
| **AGTR1** | 185 |  |  |  |  |  |  | [13] |
| **AIRE** | 326 | [14] |  |  |  |  |  |  |
| **AKT2** | 208 | [15] | [16] |  |  |  |  |  |
| **AKT3** | 10000 |  |  |  |  |  | [7] |  |
| **ALDOB** | 229 |  |  | [1] |  |  |  |  |
| **ALMS1** | 7840 | [9] | [17] |  |  |  |  |  |
| **APOD** | 347 |  |  | [11] |  |  |  |  |
| **APS** | 10603 |  |  |  |  |  | [7] |  |
| **AQP7** | 364 |  | [18] |  |  |  |  |  |
| **RHOQ** | 23433 |  |  |  |  |  | [7] |  |
| **BAD** | 572 |  |  |  |  |  | [7] |  |
| **BSCL2** | 26580 | [9] |  |  |  |  |  |  |
| **CALML6** | 163688 |  |  |  |  |  | [7] |  |
| **CAPN10** | 11132 |  |  | [9] | [12] | [6] |  |  |
| **CBLC** | 23624 |  |  |  |  |  | [7] |  |
| **CD4** | 920 |  |  | [2] |  |  |  |  |
| **CD36** | 948 |  | [19] |  |  |  | [20] |  |
| **CEL** | 1056 |  |  | [21] |  |  |  |  |
| **CP** | 1356 | [14] |  |  |  |  |  |  |
| **CRK** | 1398 |  |  |  |  |  | [7] |  |
| **CYP11A1** | 1583 | [9] |  |  |  |  |  |  |
| **CYS1** | 192668 |  |  | [12] | [12] |  |  |  |
| **DPP4** | 1803 |  | [22] |  |  |  | [23] |  |
| **EIF4E** | 1977 |  |  |  |  |  | [7] |  |
| **EIF4EBP1** | 1978 |  |  |  |  |  | [7] |  |
| **ELK1** | 2002 |  |  |  |  |  | [7] |  |
| **ENPP1** | 5167 |  |  | [9] |  |  |  |  |
| **EXOC7** | 23265 |  |  |  |  |  | [7] |  |
| **FABP2** | 2169 |  |  | [1, 11], [9] |  |  |  |  |
| **FABP3** | 2170 |  |  | [24] |  |  |  |  |
| **FASN** | 2194 |  |  |  |  |  | [7] |  |
| **FBP1** | 2203 |  |  |  |  |  | [7] |  |
| **FLOT1** | 10211 |  |  |  |  |  | [7] |  |
| **FRAP1** | 2475 |  |  |  |  |  | [5] |  |
| **FXN** | 2395 | [14] |  | [2] |  |  |  |  |
| **FOXA2** | 3170 |  | [25] |  |  |  | [26] |  |
| **FOXA3** | 3171 |  |  |  |  |  | [26] |  |
| **FOXO1A** | 2308 |  |  |  |  |  | [7] |  |
| **FOXC2** | 2303 |  | [27, 28] |  | [12] |  |  |  |
| **FUT3** | 2525 |  |  | [29] |  |  |  |  |
| **SLC37A4** | 2542 |  |  |  |  |  | [7] |  |
| **GAD2** | 2572 |  |  | [11] |  |  |  |  |
| **GC** | 2638 |  |  | [9] |  |  |  |  |
| **GCG** | 2641 |  | [30] |  |  |  | [31] |  |
| **GCGR** | 2642 |  |  | [2] |  |  |  |  |
| **GCK** | 2645 | [9] | [6] | [2] |  | [6] | [26] |  |
| **GHRL** | 51738 |  |  | [11] |  |  |  |  |
| **GIP** | 2695 |  |  |  |  |  | [32] |  |
| **GLUD1** | 2746 | [33] |  |  |  |  |  |  |
| **GNB3** | 2784 |  |  | [11] |  |  |  |  |
| **GRB2** | 2885 |  |  |  |  |  | [7] |  |
| **RASGRF2** | 2889 |  |  |  |  |  | [7] |  |
| **GYS1** | 2997 |  |  | [34] |  |  | [7] |  |
| **TPPP** | 11076 |  |  |  |  |  | [7] | [35] |
| **HES1** | 3280 |  |  |  |  |  | [26] |  |
| **HHEX** | 3087 |  |  |  |  |  | [26] |  |
| **HFE** | 3077 | [14] |  | [2] |  |  |  |  |
| **HLXB9** | 3110 |  |  |  |  |  | [26] |  |
| **TCF1** | 6927 | [9] | [6] | [2] |  | [6] | [26] |  |
| **TCF2** | 6928 | [9] |  |  |  | [6] | [26] |  |
| **HNF4A** | 3172 | [9] | [36] | [9] & [3] |  | [6] | [26] |  |
| **HNF4G** | 3174 |  |  |  |  |  | [26] |  |
| **hPDK1** | 440332 |  |  |  |  |  | [7] |  |
| **HSD11B1** | 3290 |  | [37] |  |  |  |  |  |
| **IL6** | 3569 |  |  | [2] |  |  |  |  |
| **IAPP** | 3375 |  |  | [9] |  |  | [26] |  |
| **IDE** | 3416 |  |  | [11] |  |  |  |  |
| **IGF1** | 3479 |  |  | [9] |  |  |  |  |
| **IGF1R** | 3480 |  | [36] |  |  |  |  |  |
| **IKBKB** | 3551 |  | [38] |  |  |  | [39] |  |
| **INPP5D** | 3635 |  |  |  |  |  | [7] |  |
| **INS** | 3630 | [14] |  | [9] & [3] | [12] |  | [5] |  |
| **INSR** | 3643 | [9] |  | [2] & [3] |  |  | [5] |  |
| **IPF1** | 3651 | [9] | [6] | [2] |  | [6] | [5] |  |
| **IR** | 3643 |  | [40] |  |  |  |  |  |
| **IRS1** | 3667 |  | [40] | [11] | [12] |  | [5] |  |
| **IRS2** | 8660 |  | [41] | [9] |  |  |  |  |
| **IRS4** | 8471 |  | [36] |  |  |  |  |  |
| **ISL1** | 3670 |  | [36] |  |  |  |  |  |
| **KCNJ11** | 3767 | [42] |  | [2] & [3] | [12] |  | [5] |  |
| **KLRG1** | 10219 |  |  |  |  |  | [5] |  |
| **COG2** | 22796 |  |  | [1] |  |  |  |  |
| **LEP** | 3952 |  |  | [1] |  |  |  |  |
| **LIPC** | 3990 |  |  | [3] |  |  |  |  |
| **LIPE** | 3991 |  |  | [11] |  |  | [7] |  |
| **LMNA** | 4000 | [9] |  | [11] |  |  |  |  |
| **LOC392358** | 392358 |  |  |  |  |  | [7] |  |
| **MAFA** | 389692 |  |  |  |  |  | [26] |  |
| **MAP2K1** | 5604 |  |  |  |  |  | [7] |  |
| **MAPK1** | 5594 |  |  |  |  |  | [5] |  |
| **MAPK8** | 5599 |  |  |  |  |  | [5] |  |
| **BHLHB8** | 168620 |  |  |  |  |  | [26] |  |
| **MKNK2** | 2872 |  |  |  |  |  | [7] |  |
| **MTPN** | 136319 |  |  |  |  |  | [43] |  |
| **TRNL1** | 4567 | [14] |  |  |  |  |  |  |
| **NEUROD1** | 4760 | [9] | [6] |  |  | [6] | [26] |  |
| **NEUROG3** | 50674 |  | [36] |  |  |  | [26] |  |
| **NFKB1** | 4790 |  |  |  |  |  | [39] |  |
| **NKX2-2** | 4821 |  | [6] |  |  |  | [26] |  |
| **NOS3** | 4846 |  |  | [9] |  |  |  |  |
| **NRF-1** | 4899 |  |  |  |  |  | [44] |  |
| **NROB2** | 8431 |  |  | [11] |  |  |  |  |
| **ONECUT1** | 3175 |  |  |  |  |  | [26] |  |
| **PAX4** | 5078 |  | [36] |  |  |  | [26] |  |
| **PAX6** | 5080 |  | [36] |  |  |  | [26] |  |
| **PCK1** | 5105 |  |  |  |  |  | [7] |  |
| **PCSK1** | 5122 | [14] |  |  |  |  |  |  |
| **PCSK2** | 5126 |  |  | [2] |  |  |  |  |
| **PDE3A** | 5139 |  |  |  |  |  | [7] |  |
| **PFKL** | 5211 |  |  |  |  |  | [7] |  |
| **PPARGC1A** | 10891 |  | [45, 46] | [47] | [12] |  | [48] |  |
| **PIK3R1** | 5295 |  | [36] | [9] & [3] |  |  | [5] |  |
| **PKLR** | 5313 |  |  |  |  |  | [26] |  |
| **PLAGL1** | 5325 | [49] |  |  |  |  |  |  |
| **PON2** | 5445 |  |  | [9] |  |  |  |  |
| **PPARG** | 5468 | [9] | [36] | [11] | [12] |  |  | [50] |
| **PPARGC1A** | 10891 |  |  | [11] | [51] |  | [7] |  |
| **PPP1CA** | 5499 |  |  |  |  |  | [7] |  |
| **PPP1R3A** | 5506 | [9] |  | [2] |  |  |  |  |
| **PRKACA** | 5566 |  |  |  |  |  | [7] |  |
| **PRKCE** | 5581 |  |  |  |  |  | [5] |  |
| **PRKCI** | 5584 |  |  |  |  |  | [7] |  |
| **PRKCZ** | 5590 |  |  |  |  |  | [5] |  |
| **PTF1A** | 256297 | [52] |  |  |  |  |  |  |
| **PTPN1** | 5770 |  | [36] | [53] |  |  | [7] |  |
| **PTPRF** | 5792 |  |  |  |  |  | [7] |  |
| **PYY** | 5697 |  |  | [3] |  |  |  |  |
| **PYGB** | 5834 |  |  |  |  |  | [7] |  |
| **RAF1** | 5894 |  |  |  |  |  | [7] |  |
| **RAPTOR** | 57521 |  |  |  |  |  | [54] |  |
| **RBP4** | 5950 |  | [55] |  |  |  |  |  |
| **BLM** | 641 | [9] |  |  |  |  |  |  |
| **RETN** | 56729 |  | [56] | [11] |  |  | [57] |  |
| **RHEB** | 6009 |  |  |  |  |  | [7] |  |
| **RPS6KB1** | 6198 |  |  |  |  |  | [7] |  |
| **RRAD** | 6236 |  |  | [2] |  |  |  |  |
| **RRAS** | 6237 |  |  |  |  |  | [7] |  |
| **SERPINE1** | 5054 |  |  | [11] |  |  |  |  |
| **SHC2** | 25759 |  |  |  |  |  | [7] |  |
| **SIRT1** | 23411 |  |  |  |  |  | [48] |  |
| **SIRT2** | 22933 |  |  | [58] |  |  |  |  |
| **SKIP** | 51763 |  |  |  |  |  | [7] |  |
| **SLC2A1** | 6513 |  |  | [2] | [4] |  |  |  |
| **SLC2A2** | 6514 |  | [6] | [2] |  | [6] | [5] |  |
| **SLC2A4** | 6517 |  | [6] |  |  |  | [5] |  |
| **SOCS1** | 8651 |  |  |  |  |  | [5] |  |
| **SORBS1** | 10580 |  |  | [59] |  |  | [7] |  |
| **SOS1** | 6654 |  |  |  |  |  | [7] |  |
| **SREBF1** | 6720 |  |  | [11] |  |  | [7] |  |
| **SUR1** | 6833 |  | [60] |  |  |  |  |  |
| **TCF1** | 6927 |  |  | [2] |  |  |  |  |
| **TCF7L2** | 6934 |  |  | [61] |  | [61] |  |  |
| **TGFB1** | 7040 |  |  | [11] |  |  |  |  |
| **TNF** | 7124 |  |  | [1, 11] | [62] |  | [5] |  |
| **TNFRSF1B** | 7133 |  |  | [11] |  |  |  |  |
| **CRTC2** | 200186 |  |  |  |  |  | [63] |  |
| **TSC1** | 7248 |  |  |  |  |  | [7] |  |
| **UBL5** | 59286 |  |  | [11] |  |  |  |  |
| **UCP1** | 7350 |  | [64] |  |  |  |  |  |
| **UCP2** | 7351 |  |  | [11] |  |  |  |  |
| **UCP3** | 7352 |  |  | [9] |  |  |  |  |
| **USF1** | 7391 |  |  | [65] |  | [65] |  |  |
| **WFS1** | 7466 | [14] |  |  |  |  |  |  |
| **WNT5B** | 81029 |  |  | [66] |  |  |  |  |
| **VLDLR** | 7436 |  |  | [1] |  |  |  |  |

* Evidence was recorded only for genes that lie under linkage peaks and are also implicated by other types of evidence.

References:

1.	Daimon, M., et al., *Large-scale search of SNPs for type 2 DM susceptibility genes in a Japanese population.* Biochem Biophys Res Commun, 2003. **302**(4): p. 751-8.

2.	Hirschhorn, J.N., et al., *A comprehensive review of genetic association studies.* Genet Med, 2002. **4**(2): p. 45-61.

3.	Barroso, I., et al., *Candidate gene association study in type 2 diabetes indicates a role for genes involved in beta-cell function as well as insulin action.* PLoS Biol, 2003. **1**(1): p. E20.

4.	Lohmueller, K.E., et al., *Meta-analysis of genetic association studies supports a contribution of common variants to susceptibility to common disease.* Nat Genet, 2003. **33**(2): p. 177-82.

5.	KEGG, *Type 2 Diabetes Mellitus*, in *04930*. 2006: Kyoto.

6.	Tusie Luna, M.T., *Genes and type 2 diabetes mellitus.* Arch Med Res, 2005. **36**(3): p. 210-22.

7.	KEGG, *Insulin signaling pathway*, in *04910*. 2006: Kyoto.

8.	Maeda, N., et al., *Diet-induced insulin resistance in mice lacking adiponectin/ACRP30.* Nat Med, 2002. **8**(7): p. 731-7.

9.	Barroso, I., *Genetics of Type 2 diabetes.* Diabetic Medicine, 2005. **22**(5): p. 517-535.

10.	Yamauchi, T., et al., *Cloning of adiponectin receptors that mediate antidiabetic metabolic effects.* Nature, 2003. **423**(6941): p. 762-9.

11.	Bouchard, C., et al., *Obesity Gene Map Database*. 2006.

12.	Parikh, H. and L. Groop, *Candidate Genes for Type 2 Diabetes.* Reviews in Endocrine & Metabolic Disorders, 2004. **5**(2): p. 151-176.

13.	Schupp, M., et al., *Angiotensin type 1 receptor blockers induce peroxisome proliferator-activated receptor-gamma activity.* Circulation, 2004. **109**(17): p. 2054-7.

14.	Florez, J.C., J. Hirschhorn, and D. Altshuler, *THE INHERITED BASIS OF DIABETES MELLITUS: Implications for the Genetic Analysis of Complex Traits.* Annual Review of Genomics and Human Genetics, 2003. **4**(1): p. 257-291.

15.	George, S., et al., *A family with severe insulin resistance and diabetes due to a mutation in AKT2.* Science, 2004. **304**(5675): p. 1325-8.

16.	Cho, H., et al., *Insulin resistance and a diabetes mellitus-like syndrome in mice lacking the protein kinase Akt2 (PKB beta).* Science, 2001. **292**(5522): p. 1728-31.

17.	Collin, G.B., et al., *Alms1-disrupted mice recapitulate human Alstrom syndrome.* Hum Mol Genet, 2005. **14**(16): p. 2323-33.

18.	Hibuse, T., et al., *Aquaporin 7 deficiency is associated with development of obesity through activation of adipose glycerol kinase.* Proc Natl Acad Sci U S A, 2005. **102**(31): p. 10993-8.

19.	Pravenec, M., et al., *Genetics of Cd36 and the clustering of multiple cardiovascular risk factors in spontaneous hypertension.* J Clin Invest, 1999. **103**(12): p. 1651-7.

20.	Griffin, E., et al., *A link between diabetes and atherosclerosis: Glucose regulates expression of CD36 at the level of translation.* Nat Med, 2001. **7**(7): p. 840-6.

21.	Raeder, H., et al., *Mutations in the CEL VNTR cause a syndrome of diabetes and pancreatic exocrine dysfunction.* Nat Genet, 2006. **38**(1): p. 54-62.

22.	Conarello, S.L., et al., *Mice lacking dipeptidyl peptidase IV are protected against obesity and insulin resistance.* Proc Natl Acad Sci U S A, 2003. **100**(11): p. 6825-30.

23.	Mentlein, R., *Dipeptidyl-peptidase IV (CD26)--role in the inactivation of regulatory peptides.* Regul Pept, 1999. **85**(1): p. 9-24.

24.	Shin, H.D., et al., *Polymorphisms in fatty acid-binding protein-3 (FABP3) - putative association with type 2 diabetes mellitus.* Hum Mutat, 2003. **22**(2): p. 180.

25.	Wolfrum, C., et al., *Foxa2 regulates lipid metabolism and ketogenesis in the liver during fasting and in diabetes.* Nature, 2004. **432**(7020): p. 1027-32.

26.	KEGG, *MODY*, in *04950*. 2006: Kyoto.

27.	Kim, J.K., et al., *Adipocyte-specific overexpression of FOXC2 prevents diet-induced increases in intramuscular fatty acyl CoA and insulin resistance.* Diabetes, 2005. **54**(6): p. 1657-63.

28.	Cederberg, A., et al., *FOXC2 is a winged helix gene that counteracts obesity, hypertriglyceridemia, and diet-induced insulin resistance.* Cell, 2001. **106**(5): p. 563-73.

29.	Cakir, B., et al., *Association of the Lewis genotype with cardiovascular risk factors and subclinical carotid atherosclerosis: the Atherosclerosis Risk in Communities (ARIC) study.* J Intern Med, 2004. **255**(1): p. 40-51.

30.	Knauf, C., et al., *Brain glucagon-like peptide-1 increases insulin secretion and muscle insulin resistance to favor hepatic glycogen storage.* J Clin Invest, 2005. **115**(12): p. 3554-63.

31.	Wang, Z., et al., *Glucagon-like peptide-1 is a physiological incretin in rat.* J Clin Invest, 1995. **95**(1): p. 417-21.

32.	Drucker, D.J., *The biology of incretin hormones.* Cell Metab, 2006. **3**(3): p. 153-65.

33.	Stanley, C.A., et al., *Hyperinsulinism and hyperammonemia in infants with regulatory mutations of the glutamate dehydrogenase gene.* N Engl J Med, 1998. **338**(19): p. 1352-7.

34.	Groop, L.C., et al., *Association between polymorphism of the glycogen synthase gene and non-insulin-dependent diabetes mellitus.* N Engl J Med, 1993. **328**(1): p. 10-4.

35.	Van Wauwe, J. and B. Haefner, *Glycogen synthase kinase-3 as drug target: from wallflower to center of attention.* Drug News Perspect, 2003. **16**(9): p. 557-65.

36.	Accili, D., et al., *Genetics of type 2 diabetes: insight from targeted mouse mutants.* Curr Mol Med, 2001. **1**(1): p. 9-23.

37.	Masuzaki, H., et al., *A transgenic model of visceral obesity and the metabolic syndrome.* Science, 2001. **294**(5549): p. 2166-70.

38.	Arkan, M.C., et al., *IKK-beta links inflammation to obesity-induced insulin resistance.* Nat Med, 2005. **11**(2): p. 191-8.

39.	Cai, D., et al., *Local and systemic insulin resistance resulting from hepatic activation of IKK-beta and NF-kappaB.* Nat Med, 2005. **11**(2): p. 183-90.

40.	Kahn, C.R., *Knockout mice challenge our concepts of glucose homeostasis and the pathogenesis of diabetes.* Exp Diabesity Res, 2003. **4**(3): p. 169-82.

41.	Kubota, N., et al., *Disruption of insulin receptor substrate 2 causes type 2 diabetes because of liver insulin resistance and lack of compensatory beta-cell hyperplasia.* Diabetes, 2000. **49**(11): p. 1880-9.

42.	Koster, J.C., et al., *Targeted overactivity of beta cell K(ATP) channels induces profound neonatal diabetes.* Cell, 2000. **100**(6): p. 645-54.

43.	Poy, M.N., et al., *A pancreatic islet-specific microRNA regulates insulin secretion.* Nature, 2004. **432**(7014): p. 226-30.

44.	Patti, M.E., et al., *Coordinated reduction of genes of oxidative metabolism in humans with insulin resistance and diabetes: Potential role of PGC1 and NRF1.* Proc Natl Acad Sci U S A, 2003. **100**(14): p. 8466-71.

45.	Herzig, S., et al., *CREB regulates hepatic gluconeogenesis through the coactivator PGC-1.* Nature, 2001. **413**(6852): p. 179-83.

46.	Koo, S.H., et al., *PGC-1 promotes insulin resistance in liver through PPAR-alpha-dependent induction of TRB-3.* Nat Med, 2004. **10**(5): p. 530-4.

47.	Ek, J., et al., *Mutation analysis of peroxisome proliferator-activated receptor-gamma coactivator-1 (PGC-1) and relationships of identified amino acid polymorphisms to Type II diabetes mellitus.* Diabetologia, 2001. **44**(12): p. 2220-6.

48.	Rodgers, J.T., et al., *Nutrient control of glucose homeostasis through a complex of PGC-1alpha and SIRT1.* Nature, 2005. **434**(7029): p. 113-8.

49.	Mackay, D.J., et al., *Relaxation of imprinted expression of ZAC and HYMAI in a patient with transient neonatal diabetes mellitus.* Hum Genet, 2002. **110**(2): p. 139-44.

50.	Olefsky, J.M., *Treatment of insulin resistance with peroxisome proliferator-activated receptor {gamma} agonists*

J. Clin. Invest., 2000. **106**(4): p. 467-472.

51.	Barroso, I., et al., *Meta-analysis of the Gly482Ser variant in PPARGC1A in type 2 diabetes and related phenotypes.* Diabetologia, 2006. **49**(3): p. 501-5.

52.	Sellick, G.S., et al., *Mutations in PTF1A cause pancreatic and cerebellar agenesis.* Nat Genet, 2004. **36**(12): p. 1301-5.

53.	Di Paola, R., et al., *A variation in 3' UTR of hPTP1B increases specific gene expression and associates with insulin resistance.* Am J Hum Genet, 2002. **70**(3): p. 806-12.

54.	Tzatsos, A. and K.V. Kandror, *Nutrients Suppress Phosphatidylinositol 3-Kinase/Akt Signaling via Raptor-Dependent mTOR-Mediated Insulin Receptor Substrate 1 Phosphorylation.* Mol Cell Biol, 2006. **26**(1): p. 63-76.

55.	Yang, Q., et al., *Serum retinol binding protein 4 contributes to insulin resistance in obesity and type 2 diabetes.* Nature, 2005. **436**(7049): p. 356-62.

56.	Banerjee, R.R., et al., *Regulation of fasted blood glucose by resistin.* Science, 2004. **303**(5661): p. 1195-8.

57.	Rajala, M.W., et al., *Adipose-derived resistin and gut-derived resistin-like molecule-beta selectively impair insulin action on glucose production.* J Clin Invest, 2003. **111**(2): p. 225-30.

58.	Bordone, L., et al., *Sirt1 Regulates Insulin Secretion by Repressing UCP2 in Pancreatic beta Cells.* PLoS Biol, 2005. **4**(2): p. e31.

59.	Lin, W.H., et al., *Molecular scanning of the human sorbin and SH3-domain-containing-1 (SORBS1) gene: positive association of the T228A polymorphism with obesity and type 2 diabetes.* Hum Mol Genet, 2001. **10**(17): p. 1753-60.

60.	Lam, T.K., et al., *Hypothalamic sensing of circulating fatty acids is required for glucose homeostasis.* Nat Med, 2005. **11**(3): p. 320-7.

61.	Grant, S.F., et al., *Variant of transcription factor 7-like 2 (TCF7L2) gene confers risk of type 2 diabetes.* Nat Genet, 2006.

62.	Sookoian, S.C., C. Gonzalez, and C.J. Pirola, *Meta-analysis on the G-308A tumor necrosis factor alpha gene variant and phenotypes associated with the metabolic syndrome.* Obes Res, 2005. **13**(12): p. 2122-31.

63.	Koo, S.H., et al., *The CREB coactivator TORC2 is a key regulator of fasting glucose metabolism.* Nature, 2005. **437**(7062): p. 1109-11.

64.	Ricquier, D. and L.P. Kozak, *Uncoupling Proteins*, in *Handbook of Obesity: etiology and pathophysiology*, G.A. Bray and C. Bouchard, Editors. 2004, Marcel Dekker, Inc.: New York. p. 539-558.

65.	Ng, M.C., et al., *The linkage and association of the gene encoding upstream stimulatory factor 1 with type 2 diabetes and metabolic syndrome in the Chinese population.* Diabetologia, 2005. **48**(10): p. 2018-24.

66.	Kanazawa, A., et al., *Association of the gene encoding wingless-type mammary tumor virus integration-site family member 5B (WNT5B) with type 2 diabetes.* Am J Hum Genet, 2004. **75**(5): p. 832-43.
