# Supplementary material for: Adaptations to Climate in Candidate Genes for Common Metabolic Disorders
Source: PLoS Genet. 2008 Feb 15;4(2):e32. doi: 10.1371/journal.pgen.0040032 (PMC2242814; doi:10.1371/journal.pgen.0040032)
Supplement: Supplementary file 3 [file pgen.0040032.st003.doc]

Supplementary Table 3. Literature review results for obesity.

| Gene | Gene ID | Mendelian Phenotype | Animal Model | Common Phenotype (associations) | | Linkage* | Physiology | Drug  Target |
| --- | --- | --- | --- | --- | --- | --- | --- | --- |
|  |  |  |  | Single analysis | Meta analysis |  |  |  |
| ASIP | 434 |  | [1] |  |  |  | [2] |  |
| ABCC8 | 6833 |  |  | [3] |  |  |  |  |
| ABCG5 | 64240 |  |  | [3] |  |  |  |  |
| ACACB | 32 |  | [1] |  |  |  | [2] |  |
| ADIPOQ | 9370 |  | [4] | [3] |  |  | [2] |  |
| ACACA | 31 |  |  |  |  |  | [2] |  |
| ACE | 1636 |  |  | [3] |  |  |  |  |
| ACP1 | 52 |  |  | [3] |  | [1] |  |  |
| PHACS | 84680 |  |  |  |  |  | [5] |  |
| ADA | 100 |  |  | [3] |  | [1] |  |  |
| ADCYAP1 | 116 |  | [1] |  |  |  |  |  |
| ADD1 | 118 |  |  |  |  |  | [2] |  |
| ADIPOR1 | 51094 |  |  |  |  |  | [2] |  |
| ADIPOR2 | 79602 |  |  |  |  |  | [2] |  |
| ADORA1 | 134 |  |  |  |  |  | [2] |  |
| ADORA2A | 135 |  |  |  |  |  | [2] |  |
| ADORA2B | 136 |  |  |  |  |  | [2] |  |
| ADRA1B | 147 |  | [1] |  |  |  |  |  |
| ADRA2A | 150 |  | [1] | [3] |  |  | [2] |  |
| ADRA2B | 151 |  |  | [3] |  |  |  |  |
| ADRB1 | 153 |  | [1] | [3] |  |  | [2] |  |
| ADRB2 | 154 |  | [1] | [3] |  |  | [2] |  |
| ADRB3 | 155 |  | [1] | [3] | [7] |  | [2] |  |
| ADFP | 123 |  |  |  |  |  | [2] |  |
| AEBP1 | 165 |  |  |  |  |  | [5] |  |
| AGPAT1 | 10554 |  |  |  |  |  | [2] |  |
| AGPAT2 | 10555 | [1] |  |  |  |  | [2] |  |
| AGRP | 181 |  | [1] | [3] |  |  | [8] |  |
| AGT | 183 |  |  | [3] |  |  | [2] |  |
| AHO2 | 8121 | [1] |  |  |  |  |  |  |
| AHR | 196 |  |  |  |  |  | [2] |  |
| ASRGL1 | 80150 |  | [1] |  |  |  |  |  |
| AKT1 | 207 |  | [1] |  |  |  |  |  |
| AKT2 | 208 |  | [1] |  |  |  |  |  |
| ALMS1 | 7840 | [1] | [9] |  |  |  |  |  |
| AMACR | 23600 |  | [1] |  |  |  |  |  |
| ANCR | 282 | [1] |  |  |  |  |  |  |
| ANGPTL4 | 51129 |  |  |  |  |  | [2] |  |
| ANMA | 80871 | [1] |  |  |  |  |  |  |
| APOA1 | 335 |  |  | [3] |  |  |  |  |
| APOA2 | 336 |  |  | [3] |  |  |  |  |
| APOA4 | 337 |  |  | [3] |  |  | [8] |  |
| APOB | 338 |  |  | [3] |  |  |  |  |
| APOC1 | 341 |  | [1] |  |  |  |  |  |
| APOC3 | 345 |  | [1] | [3] |  |  |  |  |
| APOD | 347 |  |  |  |  |  | [8] |  |
| APOE | 348 |  |  | [3] |  |  |  |  |
| AQP7 | 364 |  | [10] | [11] |  |  |  |  |
| AR | 367 |  | [1] | [3] |  |  | [2] |  |
| ARID5B | 84159 |  | [1] |  |  |  |  |  |
| ARNT | 405 |  |  |  |  |  | [2] |  |
| ASPA | 443 |  | [1] |  |  |  |  |  |
| ATRN | 8455 |  | [12] |  |  |  | [13] |  |
| ATP1A2 | 477 |  |  | [3] |  | [1] |  |  |
| BATF | 10538 |  | [1] |  |  |  |  |  |
| BBS1 | 582 |  | [1] |  |  |  |  |  |
| BBS1 | 582 | [1] |  |  |  |  |  |  |
| BBS2 | 583 | [1] |  |  |  |  |  |  |
| BBS3 | 84100 | [1] |  |  |  |  |  |  |
| BBS4 | 585 | [1] | [1] |  |  |  |  |  |
| BBS5 | 129880 | [1] |  |  |  |  |  |  |
| BBS7 | 55212 | [1] |  |  |  |  |  |  |
| TTC8 | 123016 | [1] |  |  |  |  |  |  |
| BDNF | 627 |  | [1] |  |  |  |  |  |
| BRS3 | 680 |  | [1] |  |  |  | [8] |  |
| BSCL2 | 26580 | [1] |  |  |  |  |  |  |
| C3 | 718 |  | [1] |  |  |  |  |  |
| CALCA | 796 |  |  |  |  |  | [8] |  |
| CLPG | 79104 |  |  |  |  |  |  |  |
| CAPN10 | 11132 |  |  | [3] |  |  |  |  |
| CART | 9607 |  | [12] | [3] |  |  | [8] |  |
| CAV1 | 857 |  | [1] |  |  |  |  |  |
| RUNX1T1 | 862 |  |  | [3] |  |  |  |  |
| CCK | 885 |  |  |  |  |  | [8] |  |
| CCKAR | 886 |  | [12] | [3] |  |  | [8] |  |
| CD36 | 948 |  | [1] |  |  |  | [2] |  |
| CDH2 | 1000 |  | [1] |  |  |  |  |  |
| CDKN1A | 1026 |  | [1] |  |  |  |  |  |
| CDKN1B | 1027 |  | [1] |  |  |  |  |  |
| CEBPA | 1050 |  | [1] |  |  |  | [2] |  |
| CEBPB | 1051 |  | [1] |  |  |  | [2] |  |
| CEBPD | 1052 |  |  |  |  |  | [2] |  |
| CHM | 1121 | [1] |  |  |  |  |  |  |
| CHRM3 | 1131 |  | [1] |  |  |  | [8] |  |
| CIDEA | 1149 |  | [1] |  |  |  |  |  |
| CLOCK | 9575 |  | [14] |  |  |  |  |  |
| CNR1 | 1268 |  | [1] |  |  |  |  |  |
| CNTF | 1270 |  | [15] |  |  |  | [15] |  |
| VPS13B | 157680 | [1] |  |  |  |  |  |  |
| COL6A2 | 1292 |  |  |  |  |  | [2] |  |
| COL8A2 | 1296 | [1] |  |  |  |  |  |  |
| CORT | 1325 |  |  |  |  |  | [8] |  |
| CPE | 1363 |  | [1] |  |  |  |  |  |
| CREB1 | 1385 |  |  |  |  |  | [2] |  |
| CRH | 1392 |  | [1] |  |  |  | [6] |  |
| CRHR1 | 1394 | [1] |  | [3] |  |  |  |  |
| CRHR2 | 1395 | [1] |  | [3] |  |  |  |  |
| CYP19A1 | 1588 |  | [1] | [3] |  |  |  |  |
| CYP7A1 | 1581 |  |  | [3] |  |  |  |  |
| DBH | 1621 |  | [1] |  |  |  | [8] |  |
| DBI | 1622 |  |  |  |  |  | [2] |  |
| DDIT3 | 1649 |  |  |  |  |  | [2] |  |
| POU3F4 | 5456 | [1] |  |  |  |  |  |  |
| DGAT1 | 8694 |  | [1] |  |  |  | [2] |  |
| DGAT2 | 84649 |  |  |  |  |  | [2] |  |
| DHCR24 | 1718 |  | [1] |  |  |  |  |  |
| DIO2 | 1734 |  |  |  |  |  | [16] |  |
| DLK1 | 8788 |  | [1] |  |  |  | [2] |  |
| DRD2 | 1813 |  |  | [3] |  |  | [8] |  |
| DRD3 | 1814 |  | [1] |  |  |  | [8] |  |
| DRD4 | 1815 |  |  | [3] |  |  | [8] |  |
| EIF4EBP1 | 1978 |  | [1] |  |  |  |  |  |
| ENPP1 | 5167 |  |  | [17] |  |  |  |  |
| ESR1 | 2099 |  | [12] | [3] |  |  | [2] |  |
| ESRRA | 2101 |  | [1] |  |  |  | [8] |  |
| ETS1 | 2113 |  |  |  |  |  | [18] |  |
| FABP2 | 2169 |  | [12] | [3] |  |  |  |  |
| FABP4 | 2167 |  | [1] |  |  |  | [2] |  |
| CFD | 1675 |  |  |  |  |  | [2] |  |
| FASN | 2194 |  |  | [3] |  |  | [2] |  |
| FGF1 | 2246 |  |  |  |  |  | [8] |  |
| FGFR3 | 2261 | [1] |  |  |  |  |  |  |
| FMR1 | 2332 | [1] |  |  |  |  |  |  |
| FOSB | 2354 |  | [1] |  |  |  |  |  |
| FOXA2 | 3170 |  | [1] |  |  |  |  |  |
| FOXC2 | 2303 |  | [1] | [3] |  |  | [2] |  |
| FOXO1A | 2308 |  | [19] |  |  |  |  |  |
| FRAP1 | 2475 |  |  |  |  |  | [20] |  |
| FSHR | 2492 |  | [1] |  |  |  |  |  |
| GABRG3 | 2567 | [1] |  |  |  |  | [8] |  |
| GAD2 | 2572 |  |  | [3] |  |  |  |  |
| GAL | 51083 |  | [1] |  |  |  | [8] |  |
| GAMT | 2593 |  | [1] |  |  |  |  |  |
| GATA2 | 2624 |  |  |  |  |  | [2] |  |
| GATA3 | 2625 |  |  |  |  |  | [2] |  |
| GCCR | 2908 |  |  |  |  |  | [2] |  |
| GCG | 2641 |  |  |  |  |  | [8] |  |
| NR3C1 | 2642 |  |  | [3] |  |  | [8] |  |
| GCK | 2645 |  | [1] | [3] |  |  |  |  |
| GDF3 | 9573 |  | [1] |  |  |  |  |  |
| GDF8 | 2660 |  | [1] |  |  |  |  |  |
| GFRA2 | 2675 |  | [1] |  |  |  |  |  |
| GH1 | 2688 | [1] |  |  |  |  | [8] |  |
| GHR | 2690 |  |  |  |  |  | [2] |  |
| GHRH | 2691 |  | [1] |  |  |  | [8] |  |
| GHRHR | 2692 | [1] |  |  |  |  | [8] |  |
| GHRL | 51738 |  | [1] | [3] |  |  | [8] |  |
| GHSR | 2693 |  | [1] |  |  |  |  |  |
| GIPR | 2696 |  | [1] |  |  |  |  |  |
| GCG | 2641 |  |  |  |  |  | [8] |  |
| GNAI1 | 2770 |  |  |  |  |  | [6] |  |
| GNAS | 2778 | [1] | [1] |  |  |  | [6] |  |
| GNB3 | 2784 |  |  | [3] |  |  |  |  |
| GNG3 | 2785 |  | [1] |  |  |  |  |  |
| GNRH1 | 2796 |  |  |  |  |  | [8] |  |
| GPAM | 57678 |  | [1] |  |  |  |  |  |
| GPC3 | 2719 | [1] |  |  |  |  |  |  |
| GPC4 | 2239 | [1] |  |  |  |  |  |  |
| GPD1 | 2819 |  |  |  |  |  | [18] |  |
| GPD2 | 2820 |  | [1] |  |  |  | [2] |  |
| GPR24 | 2847 | [1] | [1] |  |  |  |  |  |
| GPR7 | 2831 |  | [1] |  |  |  |  |  |
| GPX1 | 2876 |  | [1] |  |  |  |  |  |
| GSK3B | 2932 |  | [1] |  |  |  |  |  |
| GTRGEO22 | 91978 |  | [1] |  |  |  |  |  |
| H6PD | 9563 | [1] |  |  |  |  |  |  |
| HCRT | 3060 |  | [1] |  |  |  | [8] |  |
| HCRTR1 | 3061 |  |  |  |  |  | [8] |  |
| HCRTR2 | 3062 |  |  |  |  |  | [8] |  |
| HD | 3067 |  | [1] |  |  |  |  |  |
| HDH | 25939 |  | [1] |  |  |  |  |  |
| HMGA2 | 8091 |  | [1] |  |  |  | [2] |  |
| HRH1 | 3269 |  | [1] |  |  |  |  |  |
| HRH3 | 11255 |  | [1] |  |  |  |  |  |
| HSD11B1 | 3290 | [1] | [1] | [3] |  |  | [5] |  |
| HSD3B1 | 3283 |  |  | [3] |  |  |  |  |
| HSPA1B | 3304 |  |  | [3] |  |  |  |  |
| HTR1B | 3351 |  | [12] | [3] |  |  | [8] |  |
| HTR2A | 3356 |  |  | [3] |  |  | [8] |  |
| HTR2C | 3358 |  | [1] | [3] |  |  | [8] |  |
| IAPP | 3375 |  |  |  |  |  | [8] |  |
| ICAM1 | 3383 |  | [1] |  |  |  | [2] |  |
| IDE | 3416 |  |  | [3] |  |  |  |  |
| IDH1 | 3417 |  | [1] |  |  |  |  |  |
| ID2 | 3398 |  |  |  |  |  | [2] |  |
| ID3 | 3399 |  |  |  |  |  | [2] |  |
| IGF1 | 3479 |  |  | [3] |  |  | [2] |  |
| IGF2 | 3481 |  |  | [3] |  |  |  |  |
| IKBKB | 3551 |  | [12] |  |  |  |  |  |
| IL1A | 3552 |  |  |  |  |  | [8] |  |
| IL1RN | 3557 |  | [1] |  |  |  | [8] |  |
| IL6 | 3569 |  | [1] | [3] |  |  | [8] |  |
| IL6R | 3570 |  |  | [3] |  |  | [8] |  |
| INS | 3630 |  |  | [3] |  |  | [5] |  |
| INSIG1 | 3638 | [21] |  |  |  |  |  |  |
| INSR | 3643 | [1] | [1] |  |  |  | [5] |  |
| IPW | 3653 | [1] |  |  |  |  |  |  |
| IRS1 | 3667 |  |  | [3] |  |  | [6] |  |
| IRS2 | 8660 |  | [1] |  |  |  | [6] |  |
| JUN | 3725 |  |  |  |  |  | [18] |  |
| KCNA3 | 3738 |  | [1] |  |  |  |  |  |
| KCNJ11 | 3767 |  | [1] |  |  |  |  |  |
| KL | 9365 |  | [12] |  |  |  |  |  |
| LDLR | 3949 |  |  | [3] |  |  |  |  |
| LEP | 3952 | [1] | [1] | [3] |  | [1] | [2] |  |
| LEPR | 3953 | [1] | [1] | [3] |  | [1] | [8] |  |
| LHB | 3972 |  | [1] |  |  |  |  |  |
| LIFR | 3977 |  |  |  |  |  | [2] |  |
| LIPC | 3990 |  | [1] | [3] |  |  |  |  |
| LIPE | 3991 |  | [1] | [3] |  |  | [2] |  |
| LMNA | 4000 | [1] |  | [3] |  |  |  |  |
| LPIN1 | 23175 |  | [1] |  |  |  |  |  |
| LPL | 4023 |  |  | [3] |  |  | [2] |  |
| LRPAP1 | 4043 |  |  | [3] |  |  |  |  |
| ITGAM | 3684 |  |  |  |  |  | [2] |  |
| MAGEL2 | 54551 | [1] |  |  |  |  |  |  |
| MAOA | 4128 |  |  | [3] |  |  |  |  |
| MAPK8IP1 | 9479 |  | [1] |  |  |  |  |  |
| MC3R | 4159 | [1] | [1] | [3] |  |  | [8] |  |
| MC4R | 4160 | [1] | [1] | [3] |  | [1] | [8] |  |
| MECP2 | 4204 | [1] |  |  |  |  |  |  |
| MEHMO | 8422 | [1] |  |  |  |  |  |  |
| MEN1 | 4221 | [1] |  |  |  |  |  |  |
| MIF | 4282 |  |  | [22] |  |  |  |  |
| MKKS | 8195 | [1] |  |  |  |  |  |  |
| MKRN3 | 7681 | [1] |  |  |  |  |  |  |
| MMP19 | 4327 |  | [1] |  |  |  |  |  |
| MRXS11 | 50644 | [1] |  |  |  |  |  |  |
| MRXS7 | 4429 | [1] |  |  |  |  |  |  |
| MT1A | 4489 |  | [1] |  |  |  | [2] |  |
| MYC | 4609 |  | [1] |  |  |  |  |  |
| NCB5OR | 51167 |  | [1] |  |  |  |  |  |
| NDN | 4692 | [1] |  |  |  |  |  |  |
| NHLH2 | 4808 |  | [1] |  |  |  |  |  |
| NMU | 10874 |  | [1] |  |  |  | [8] |  |
| NPPA | 4878 |  |  |  |  |  | [6] |  |
| NPPB | 4879 |  |  |  |  |  | [6] |  |
| NPR1 | 4881 |  |  |  |  |  | [6] |  |
| NPR3 | 4883 |  |  | [3] |  |  |  |  |
| NPY | 4852 |  | [1] | [3] |  | [1] | [8] |  |
| NPY1R | 4886 |  | [1] |  |  |  | [6] |  |
| NPY2R | 4887 |  |  | [3] |  |  | [6] |  |
| NPY5R | 4889 |  | [1] | [3] |  |  | [6] |  |
| NR5A1 | 2516 |  | [12] |  |  |  |  |  |
| NR3C1 | 2908 |  | [1] | [3] |  | [1] |  |  |
| NR2F1 | 7025 |  |  |  |  |  | [5] |  |
| NRF1 | 4899 |  |  |  |  |  | [16] |  |
| NRF2 | 4780 |  |  |  |  |  | [16] |  |
| NRLI3 | 9970 |  | [1] |  |  |  |  |  |
| NR0B2 | 8431 |  |  | [3] |  |  |  |  |
| NTS | 4922 |  |  |  |  |  | [8] |  |
| NTSR1 | 4923 |  | [1] |  |  |  | [8] |  |
| OXT | 5020 |  |  |  |  |  | [8] |  |
| OXTR | 5021 |  |  |  |  |  | [8] |  |
| SERPINE1 | 5054 |  | [12] |  |  |  | [2] |  |
| PARP1 | 142 |  | [1] |  |  |  |  |  |
| PAX6 | 5080 | [1] |  |  |  |  |  |  |
| PCK1 | 5105 |  |  |  |  |  | [2] |  |
| PCSK1 | 5122 | [1] | [1] |  |  |  |  |  |
| PCSK1N | 27344 |  | [1] |  |  |  |  |  |
| PDE3B | 5140 |  |  |  |  |  | [6] |  |
| PHF6 | 84295 | [1] |  |  |  |  |  |  |
| PIK3R1 | 5295 |  | [1] |  |  |  | [6] |  |
| PLA2G1B | 5319 |  | [1] |  |  |  |  |  |
| PIP5K2B | 8396 |  | [1] |  |  |  |  |  |
| PLIN | 5346 |  | [1] | [3] |  |  | [2] |  |
| PLSCR1 | 5359 |  | [1] |  |  |  |  |  |
| PLSCR3 | 57048 |  | [1] |  |  |  |  |  |
| PMCH | 5367 |  | [1] |  |  |  |  |  |
| PMM2 | 5373 | [1] |  |  |  |  |  |  |
| PNMT | 5409 |  |  | [3] |  |  |  |  |
| POMC | 5443 | [1] | [1] |  |  |  | [6] |  |
| PON2 | 5445 |  |  | [3] |  |  |  |  |
| PPARA | 5465 |  | [1] | [3] |  |  |  |  |
| PPARD | 5467 |  | [1] | [3] |  |  | [2] |  |
| PPARG | 5468 | [1] | [1] | [3] | [24] |  | [2] |  |
| PPARGC1A | 10891 |  |  | [3] |  |  | [16] |  |
| PPARGC1B | 133522 |  | [1] |  |  |  |  |  |
| PRLH | 51052 |  |  |  |  |  | [8] |  |
| PPP1R3A | 5506 | [1] |  |  |  |  |  |  |
| PPP1R3C | 5507 | [25] | [1] |  |  |  |  |  |
| PPY | 5539 |  | [1] |  |  |  |  |  |
| PYY | 5697 |  |  |  |  |  | [6] |  |
| PRKAA2 | 5563 |  | [1] |  |  |  | [6] |  |
| PRKACA | 5566 |  |  |  |  |  | [16] |  |
| PRKACB | 5567 |  |  |  |  |  | [16] |  |
| PRKAB1 (aka AMPK) | 5564 |  | [26] |  |  |  | [6] |  |
| PRKAG3 | 53632 |  | [1] |  |  |  | [6] |  |
| PRKAR1A | 5573 | [1] |  |  |  |  | [6] |  |
| PRKAR2B | 5577 |  | [1] |  |  |  | [2] |  |
| PRKCQ | 5588 |  | [1] |  |  |  |  |  |
| PRLR | 5618 |  |  |  |  |  | [2] |  |
| PROP1 | 5626 | [1] |  |  |  |  |  |  |
| PTGIR | 5739 |  |  |  |  |  | [2] |  |
| PTGER1 | 5731 |  |  |  |  |  | [2] |  |
| PTGER3 | 5733 |  |  |  |  |  | [2] |  |
| PTGER4 | 5734 |  |  |  |  |  | [2] |  |
| PTGFR | 5737 |  |  |  |  |  | [2] |  |
| PTGS2 | 5743 |  | [12] |  |  |  |  |  |
| PTPN1 | 5770 |  | [1] | [3] |  |  | [2] |  |
| PTPRF | 5792 |  |  | [3] |  |  |  |  |
| PTTG1 | 9232 |  | [1] |  |  |  |  |  |
| PWCR1 | 63968 | [1] |  |  |  |  |  |  |
| RAB3GAP1 | 22930 |  |  |  |  |  | [5] |  |
| RAPTOR | 57521 |  |  |  |  |  | [20] |  |
| RARA | 5914 |  |  |  |  |  | [2] |  |
| RARG | 5916 |  |  |  |  |  | [2] |  |
| RASGRF1 | 5923 |  | [1] |  |  |  |  |  |
| RB1 | 5925 |  |  |  |  |  | [5] |  |
| RBL1 | 5933 |  |  |  |  |  | [5] |  |
| REN | 5972 |  | [1] |  |  |  |  |  |
| RETN | 56729 |  | [1] | [3] |  |  | [2] |  |
| RPS6KA3 | 6197 |  | [1] |  |  |  |  |  |
| RPS6KB1 | 6198 |  | [1] |  |  |  |  |  |
| RXRA | 6256 |  |  |  |  |  | [2] |  |
| RXRB | 6257 |  |  |  |  |  | [2] |  |
| RXRG | 6258 |  | [1] |  |  |  | [16] |  |
| ACSM3 | 6296 |  |  | [3] |  |  |  |  |
| SCARB1 | 949 |  |  | [3] |  |  |  |  |
| SCD | 6319 |  | [1] |  |  |  |  |  |
| SDC1 | 6382 |  | [1] |  |  |  |  |  |
| SDC3 | 9672 |  | [1] |  |  |  |  |  |
| SERPINE1 | 5054 |  | [1] | [3] |  |  |  |  |
| SFRP1 | 6422 |  | [1] |  |  |  |  |  |
| SGBS2 | 23402 | [1] |  |  |  |  |  |  |
| PTPN11 | 5781 |  |  |  |  |  | [8] |  |
| SIM1 | 6492 | [1] | [1] |  |  |  |  |  |
| SLC2A2 | 6514 | [1] |  |  |  |  | [8] |  |
| SLC2A4 | 6517 |  | [1] |  |  |  | [2] |  |
| SLC6A2 | 6530 |  |  |  |  |  | [8] |  |
| SLC6A3 | 6531 |  | [12] |  |  |  |  |  |
| SLC6A14 | 11254 |  | [1] | [3] |  |  |  |  |
| SLC27A1 | 376497 |  |  |  |  |  | [5] |  |
| SLC27A4 | 10999 |  |  |  |  |  | [27] |  |
| SNRPN | 6638 | [1] |  |  |  |  |  |  |
| SOCS3 | 9021 |  | [1] |  |  |  | [8] |  |
| SORBS1 | 10580 |  |  | [3] |  |  |  |  |
| SPARC | 6678 |  | [1] |  |  |  |  |  |
| SREBF1 | 6720 |  | [1] | [3] |  |  | [2] |  |
| STAT1 | 6772 |  |  |  |  |  | [5] |  |
| STAT3 | 6774 |  | [1] |  |  |  | [5] |  |
| STAT5A | 6776 |  |  |  |  |  | [5] |  |
| STAT5B | 6777 |  | [1] |  |  |  | [5] |  |
| STK25 | 10494 | [1] |  |  |  |  |  |  |
| TBX3 | 6926 | [1] |  |  |  |  |  |  |
| TCF4 | 6925 |  |  |  |  |  | [5] |  |
| TFAP2A | 7020 |  |  |  |  |  | [2] |  |
| TGFB1 | 7040 |  | [1] | [3] |  |  |  |  |
| THRA | 7067 |  | [1] |  |  |  | [2] |  |
| THRB | 7068 | [1] |  |  |  |  | [2] |  |
| TKT | 7086 |  | [1] |  |  |  |  |  |
| TNF | 7124 |  | [1] | [3] | [28] | [1] | [5] |  |
| TNFRSF1B | 7133 |  | [12] | [3] |  |  | [8] |  |
| TRH | 7200 |  |  |  |  |  | [8] |  |
| TUB | 7275 |  | [1] |  |  |  |  |  |
| UBL5 | 59286 |  |  | [3] |  |  | [8] |  |
| UCP1 | 7350 |  | [1] | [3] |  |  | [2] |  |
| UCP2 | 7351 |  |  | [3] |  |  | [16] |  |
| UCP3 | 7352 |  | [1] | [3] |  |  | [16] |  |
| UNC5C | 8633 |  | [1] |  |  |  |  |  |
| PDYN | 5173 |  |  |  |  |  | [8] |  |
| VDR | 7421 |  |  | [3] |  |  |  |  |
| VGF | 7425 |  | [1] |  |  |  |  |  |
| VLDLR | 7436 |  | [12] |  |  |  | [2] |  |
| VSX1 | 30813 | [1] |  |  |  |  |  |  |
| VSX1 | 30813 | [1] |  |  |  |  |  |  |
| WNT1 | 7471 |  |  |  |  |  | [5] |  |
| WNT5B | 81029 |  | [29] |  |  |  | [5] |  |
| WNT10B | 7480 |  | [1] |  |  |  |  |  |
| WT1 | 7490 | [1] |  |  |  |  |  |  |
| WTS | 7492 | [1] |  |  |  |  |  |  |
| ZFP36 | 7538 |  | [1] |  |  |  |  |  |
| MKRN3 | 7681 | [1] |  |  |  |  |  |  |

* Evidence was recorded only for genes that lie under linkage peaks and are also implicated by other types of evidence.

References:

1.	Perusse, L., et al., *The human obesity gene map: the 2004 update.* Obes Res, 2005. **13**(3): p. 381-490.

2.	Ailhaud, G. and H. Hauner, *Development of White Adipose Tissue*, in *Handbook of Obesity: etiology and pathophysiology*, G.A. Bray and C. Bouchard, Editors. 2004, Marcel Dekker, Inc.: New York. p. 481-514.

3.	Bouchard, C., et al., *Obesity Gene Map Database*. 2006.

4.	Shklyaev, S., et al., *Sustained peripheral expression of transgene adiponectin offsets the development of diet-induced obesity in rats.* Proc Natl Acad Sci U S A, 2003. **100**(24): p. 14217-22.

5.	Fajas, L. and J. Auwerx, *Peroxisome proliferator-activated receptor gamma and the transcriptional control of adipogenesis and metabolism*, in *Handbook of Obesity: etiology and pathophysiology*, G.A. Bray and C. Bouchard, Editors. 2004, Marcel Dekker, Inc.: New York. p. 559-587.

6.	Langin, D. and M. Lafontan, *Lipolysis and lipid mobilization in human adipose tissue*, in *Handbook of Obesity: etiology and pathophysiology*, G.A. Bray and C. Bouchard, Editors. 2004, Marcel Dekker, Inc.: New York. p. 515-532.

7.	Kurokawa, N., et al., *Association of BMI with the beta3-adrenergic receptor gene polymorphism in Japanese: meta-analysis.* Obes Res, 2001. **9**(12): p. 741-5.

8.	Leibowitz, S.F. and B.G. Hoebel, *Behavioral Neuroscience and Obesity*, in *Handbook of Obesity: etiology and pathophysiology*, G.A. Bray and C. Bouchard, Editors. 2004, Marcel Dekker, Inc.: New York. p. 301-371.

9.	Collin, G.B., et al., *Alms1-disrupted mice recapitulate human Alstrom syndrome.* Hum Mol Genet, 2005. **14**(16): p. 2323-33.

10.	Hara-Chikuma, M., et al., *Progressive adipocyte hypertrophy in aquaporin-7-deficient mice: adipocyte glycerol permeability as a novel regulator of fat accumulation.* J Biol Chem, 2005. **280**(16): p. 15493-6.

11.	Hibuse, T., et al., *Aquaporin 7 deficiency is associated with development of obesity through activation of adipose glycerol kinase.* Proc Natl Acad Sci U S A, 2005. **102**(31): p. 10993-8.

12.	Allison, D.B., et al., *Genetic Influences on Obesity*, in *Obesity: Mechanisms and Clinical Management*, R. Eckel, Editor. 2003, Elsevier: New York. p. 31-74.

13.	He, L., et al., *A biochemical function for attractin in agouti-induced pigmentation and obesity.* Nat Genet, 2001. **27**(1): p. 40-7.

14.	Turek, F.W., et al., *Obesity and metabolic syndrome in circadian Clock mutant mice.* Science, 2005. **308**(5724): p. 1043-5.

15.	Gloaguen, I., et al., *Ciliary neurotrophic factor corrects obesity and diabetes associated with leptin deficiency and resistance.* Proc Natl Acad Sci U S A, 1997. **94**(12): p. 6456-61.

16.	Lowell, B.B. and B.M. Spiegelman, *Towards a molecular understanding of adaptive thermogenesis.* Nature, 2000. **404**(6778): p. 652-60.

17.	Matsuoka, N., et al., *Association of K121Q polymorphism in ENPP1 (PC-1) with BMI in Caucasian and African-American adults.* Int J Obes (Lond), 2006. **30**(2): p. 233-7.

18.	Ricquier, D. and L.P. Kozak, *Uncoupling Proteins*, in *Handbook of Obesity: etiology and pathophysiology*, G.A. Bray and C. Bouchard, Editors. 2004, Marcel Dekker, Inc.: New York. p. 539-558.

19.	Kamei, Y., et al., *Skeletal muscle FOXO1 (FKHR) transgenic mice have less skeletal muscle mass, down-regulated Type I (slow twitch/red muscle) fiber genes, and impaired glycemic control.* J Biol Chem, 2004. **279**(39): p. 41114-23.

20.	Tzatsos, A. and K.V. Kandror, *Nutrients Suppress Phosphatidylinositol 3-Kinase/Akt Signaling via Raptor-Dependent mTOR-Mediated Insulin Receptor Substrate 1 Phosphorylation.* Mol Cell Biol, 2006. **26**(1): p. 63-76.

21.	Li, J., et al., *Insig-1 "brakes" lipogenesis in adipocytes and inhibits differentiation of preadipocytes.* Proc Natl Acad Sci U S A, 2003. **100**(16): p. 9476-81.

22.	Sakaue, S., et al., *Promoter polymorphism in the macrophage migration inhibitory factor gene is associated with obesity.* Int J Obes (Lond), 2006. **30**(2): p. 238-42.

23.	Qu, D., et al., *A role for melanin-concentrating hormone in the central regulation of feeding behaviour.* Nature, 1996. **380**(6571): p. 243-7.

24.	Masud, S. and S. Ye, *Effect of the peroxisome proliferator activated receptor-gamma gene Pro12Ala variant on body mass index: a meta-analysis.* J Med Genet, 2003. **40**(10): p. 773-80.

25.	Snyder, E.E., et al., *The human obesity gene map: the 2003 update.* Obes Res, 2004. **12**(3): p. 369-439.

26.	Minokoshi, Y., et al., *AMP-kinase regulates food intake by responding to hormonal and nutrient signals in the hypothalamus.* Nature, 2004. **428**(6982): p. 569-74.

27.	Moulson, C.L., et al., *Cloning of wrinkle-free, a previously uncharacterized mouse mutation, reveals crucial roles for fatty acid transport protein 4 in skin and hair development.* Proc Natl Acad Sci U S A, 2003. **100**(9): p. 5274-9.

28.	Sookoian, S.C., C. Gonzalez, and C.J. Pirola, *Meta-analysis on the G-308A tumor necrosis factor alpha gene variant and phenotypes associated with the metabolic syndrome.* Obes Res, 2005. **13**(12): p. 2122-31.

29.	Kanazawa, A., et al., *Association of the gene encoding wingless-type mammary tumor virus integration-site family member 5B (WNT5B) with type 2 diabetes.* Am J Hum Genet, 2004. **75**(5): p. 832-43.
