# Supplementary material for: Adaptations to Climate in Candidate Genes for Common Metabolic Disorders
Source: PLoS Genet. 2008 Feb 15;4(2):e32. doi: 10.1371/journal.pgen.0040032 (PMC2242814; doi:10.1371/journal.pgen.0040032)
Supplement: Supplementary file 4 [file pgen.0040032.st004.doc]

Supplementary Table 4. Literature review results for Dyslipidemia.

| Gene | GeneID | Mendelian phenotype | Animal model | Common phenotype (associations) | | Linkage* | Physiology | Drug target |
| --- | --- | --- | --- | --- | --- | --- | --- | --- |
|  |  |  |  | Single analysis | Meta analysis |  |  |  |
| ABCA1 | 19 | [1] | [2] | [3] & [4] |  |  | [5] |  |
| ABCG5 | 64240 | [6] | [7] |  |  |  | [5] |  |
| ABCG8 | 64241 | [8] | [9] |  |  |  | [10] |  |
| ACAT1 | 38 |  |  |  |  |  | [5] |  |
| ADIPOQ | 9370 |  | [11] |  |  |  |  |  |
| APOA1 | 335 | [12] | [9] | [13] |  |  | [5] |  |
| APOA2 | 336 |  | [9] | [14] |  |  | [5] |  |
| APOA4 | 337 |  | [9] | [13] |  |  | [5] |  |
| APOA5 | 116519 |  | [9] | [13] |  |  |  |  |
| APOB | 338 | [15] | [16] | [13] | [17] | [18] | [5] |  |
| APOBEC1 | 339 |  |  |  |  |  | [5] |  |
| APOC1 | 341 |  | [19] |  |  |  | [5] |  |
| APOC2 | 344 | [20] & [21] |  |  |  |  | [5] |  |
| APOC3 | 345 |  | [9] | [13] |  |  | [5] |  |
| APOD | 347 |  |  |  |  |  | [5] |  |
| APOE | 348 | [22] | [9] | [13] | [23] | [24] | [5] |  |
| APOH | 350 |  |  | [25] |  |  | [5] |  |
| CLU | 1191 |  |  |  |  |  | [5] |  |
| APOL1 | 8542 |  |  |  |  |  | [5] |  |
| LDLRAP1 | 26119 | [26] |  |  |  |  | [5] |  |
| AQP7 | 364 |  | [27] |  |  |  |  |  |
| ASIP | 434 |  |  |  |  |  | [5] |  |
| CD36 | 948 |  | [28] |  |  |  | [5] |  |
| CEBPA | 1050 |  | [29] |  |  |  |  |  |
| CEL | 1056 |  |  |  |  |  | [5] |  |
| CETP | 1071 |  | [9] | [13] | [30] |  | [5] |  |
| CLOCK | 9575 |  | [31] |  |  |  |  |  |
| CUBN | 8029 |  |  |  |  |  | [5] |  |
| CYP7A1 | 1581 |  |  | [13] |  |  | [5] |  |
| CYP27A1 | 1593 |  |  |  |  |  | [5] |  |
| FABP1 | 2168 |  |  | [13] |  |  | [5] |  |
| FABP2 | 2169 |  |  | [13] |  |  | [5] |  |
| FASN | 2194 |  |  |  |  |  | [5] |  |
| FOXO1A | 2308 |  | [32] |  |  |  |  |  |
| HMGCR | 3156 |  |  | [33] |  |  | [5] | [34] |
| HMGCS1 | 3157 |  |  |  |  |  | [5] |  |
| HNF4A | 3172 |  | [9] |  |  |  |  |  |
| HSD11B1 | 3290 |  | [35] |  |  |  |  |  |
| INSIG1 | 3638 |  | [36] |  |  |  |  |  |
| INSIG2 | 51141 |  | [36] & [37] |  |  |  |  |  |
| LCAT | 3931 | [38] | [9] | [39] |  |  | [5] |  |
| LDLR | 3949 | [13] |  | [13] |  |  | [5] |  |
| LDLRAP1 | 26119 | [40] |  |  |  |  | [41] |  |
| LIPA | 3988 |  |  |  |  |  | [5] |  |
| LIPC | 3990 |  | [9] | [13] |  |  | [5] |  |
| LIPE | 3991 |  | [9] | [42] |  |  | [5] |  |
| LIPG | 9388 |  | [9] | [13] |  |  |  |  |
| LIPI | 149998 |  | [43] | [43] |  |  |  |  |
| LMNA | 4000 | [44] |  | [42] |  |  | [5] |  |
| LPA | 4018 |  | [45] | [46] & [47] |  |  | [5] |  |
| LPL | 4023 | [48] | [9] | [42] & [13] | [49] | [50] | [5] |  |
| LRP1 | 4035 |  |  |  |  |  | [5] |  |
| LRP8 | 7804 |  |  |  |  |  | [5] |  |
| LRPAP1 | 4043 |  |  |  |  |  | [5] |  |
| NR1H3 | 10062 |  | [51] |  |  |  |  |  |
| MAP17 | 10158 |  | [9] |  |  |  |  |  |
| MSR1 | 4481 |  |  |  |  |  | [5] |  |
| MTTP | 4547 | [52] | [52] | [13] |  |  | [5] |  |
| NPC1 | 4864 |  |  |  |  |  | [5] |  |
| NPC1L1 | 29881 |  | [53] | [54] |  |  | [53] |  |
| NFKB1 | 4790 |  | [9] |  |  |  |  |  |
| NPY | 4852 |  |  | [55] |  |  |  |  |
| NR1H4 | 9971 |  | [9] |  |  |  |  | [56] |
| PCSK9 | 255738 | [57] | [58] |  |  |  |  |  |
| PCYT1A | 5130 |  | [9] |  |  |  |  |  |
| PDZK1 | 5174 |  | [9] |  |  |  |  |  |
| PEMT | 10400 |  | [9] |  |  |  |  |  |
| PLTP | 5360 |  | [9] |  |  |  | [5] |  |
| PNLIP | 5406 |  |  |  |  |  | [5] |  |
| PNPLA2 | 57104 |  |  |  |  |  | [59] |  |
| PON1 | 5444 |  | [60] | [13] |  |  | [5] |  |
| PON2 | 5445 |  |  | [61] & [62] |  |  |  |  |
| PPARA | 5465 |  | [9] | [13] |  |  | [5] | [63] |
| PPARD | 5467 |  | [64] |  |  |  | [5] |  |
| PPARG | 5468 | [65] |  | [42] & [13] |  |  | [5] |  |
| PPARGC1A | 10891 |  |  | [42] |  |  |  |  |
| RXRA | 6256 |  |  |  |  |  | [5] |  |
| ACSM3 | 6296 |  |  | [42] |  |  |  |  |
| SAA1 | 6288 |  |  |  |  |  | [5] |  |
| SAA2 | 6289 |  |  |  |  |  | [5] |  |
| SAA4 | 6291 |  |  |  |  |  | [5] |  |
| SCARB1 | 949 |  | [9] | [13] |  |  | [5] |  |
| SCAP | 22937 |  | [66] |  |  |  | [67] |  |
| SCD | 6319 |  |  |  |  |  | [5] |  |
| SCP2 | 6342 |  |  |  |  |  | [5] |  |
| SLC22A5 | 6584 |  |  |  |  |  | [5] |  |
| SREBF1 | 6720 |  | [68] |  |  |  | [69] |  |
| SREBF2 | 6721 |  | [70] |  |  |  | [69] |  |
| TCF1 | 6927 |  | [9] | [71] |  |  |  |  |
| TNFRSF1B | 7133 |  |  | [42] | [72] |  |  |  |
| UBL5 | 59286 |  |  | [42] |  |  |  |  |
| UCP1 | 7350 |  |  | [42] |  |  |  |  |
| USF1 | 7391 |  |  | [73] |  | [74] | [26] |  |
| VLDLR | 7436 |  |  |  |  |  | [5] |  |

* Evidence was recorded only for genes that lie under linkage peaks and are also implicated by other types of evidence.

References:

1.	Bodzioch, M., et al., *The gene encoding ATP-binding cassette transporter 1 is mutated in Tangier disease.* Nat Genet, 1999. 22(4): p. 347-51.

2.	McNeish, J., et al., *High density lipoprotein deficiency and foam cell accumulation in mice with targeted disruption of ATP-binding cassette transporter-1.* Proc Natl Acad Sci U S A, 2000. 97(8): p. 4245-50.

3.	Shioji, K., et al., *A promoter variant of the ATP-binding cassette transporter A1 gene alters the HDL cholesterol level in the general Japanese population.* J Hum Genet, 2004. 49(3): p. 141-7.

4.	Yamakawa-Kobayashi, K., et al., *Associations between serum high-density lipoprotein cholesterol or apolipoprotein AI levels and common genetic variants of the ABCA1 gene in Japanese school-aged children.* Metabolism, 2004. 53(2): p. 182-6.

5.	Benlian, P., *Genetics of Dyslipidemia*. 2001, Kluwer Academic Publishers.

6.	Lee, M.H., et al., *Identification of a gene, ABCG5, important in the regulation of dietary cholesterol absorption.* Nat Genet, 2001. 27(1): p. 79-83.

7.	Yang, C., et al., *Disruption of cholesterol homeostasis by plant sterols.* J Clin Invest, 2004. 114(6): p. 813-22.

8.	Berge, K.E., et al., *Accumulation of dietary cholesterol in sitosterolemia caused by mutations in adjacent ABC transporters.* Science, 2000. 290(5497): p. 1771-5.

9.	Wang, X. and B. Paigen, *Genetics of variation in HDL cholesterol in humans and mice.* Circ Res, 2005. 96(1): p. 27-42.

10.	Lusis, A.J., A.M. Fogelman, and G.C. Fonarow, *Genetic basis of atherosclerosis: part I: new genes and pathways.* Circulation, 2004. 110(13): p. 1868-73.

11.	Maeda, N., et al., *Diet-induced insulin resistance in mice lacking adiponectin/ACRP30.* Nat Med, 2002. 8(7): p. 731-7.

12.	Nakata, K., et al., *Autosomal dominant hypoalphalipoproteinemia due to a completely defective apolipoprotein A-I gene.* Biochem Biophys Res Commun, 1993. 196(2): p. 950-5.

13.	Corella, D. and J.M. Ordovas, *SINGLE NUCLEOTIDE POLYMORPHISMS THAT INFLUENCE LIPID METABOLISM: Interaction with Dietary Factors.* Annu Rev Nutr, 2005. 25: p. 341-90.

14.	Rajput-Williams, J., et al., *Plasma lipoprotein lipids in relation to the MspI polymorphism of the apolipoprotein AII gene in Caucasian men. Lack of association with plasma triglyceride concentration.* Atherosclerosis, 1989. 77(1): p. 31-6.

15.	Young, S.G., et al., *Genetic analysis of a kindred with familial hypobetalipoproteinemia. Evidence for two separate gene defects: one associated with an abnormal apolipoprotein B species, apolipoprotein B-37; and a second associated with low plasma concentrations of apolipoprotein B-100.* J Clin Invest, 1987. 79(6): p. 1842-51.

16.	Callow, M.J., et al., *Atherogenesis in transgenic mice with human apolipoprotein B and lipoprotein (a).* J Clin Invest, 1995. 96(3): p. 1639-46.

17.	Chiodini, B.D., et al., *APO B gene polymorphisms and coronary artery disease: a meta-analysis.* Atherosclerosis, 2003. 167(2): p. 355-66.

18.	Pulai, J.I., et al., *Genetic heterogeneity in familial hypobetalipoproteinemia: linkage and non-linkage to the apoB gene in Caucasian families.* Am J Med Genet, 1998. 76(1): p. 79-86.

19.	Jong, M.C., et al., *Reversal of hyperlipidaemia in apolipoprotein C1 transgenic mice by adenovirus-mediated gene delivery of the low-density-lipoprotein receptor, but not by the very-low-density-lipoprotein receptor.* Biochem J, 1999. 338 ( Pt 2): p. 281-7.

20.	Fojo, S.S., et al., *A nonsense mutation in the apolipoprotein C-IIPadova gene in a patient with apolipoprotein C-II deficiency.* J Clin Invest, 1989. 84(4): p. 1215-9.

21.	Connelly, P.W., G.F. Maguire, and J.A. Little, *Apolipoprotein CIISt. Michael. Familial apolipoprotein CII deficiency associated with premature vascular disease.* J Clin Invest, 1987. 80(6): p. 1597-606.

22.	Sullivan, P.M., et al., *Type III hyperlipoproteinemia and spontaneous atherosclerosis in mice resulting from gene replacement of mouse Apoe with human Apoe*2.* J Clin Invest, 1998. 102(1): p. 130-5.

23.	Song, Y., M.J. Stampfer, and S. Liu, *Meta-analysis: apolipoprotein E genotypes and risk for coronary heart disease.* Ann Intern Med, 2004. 141(2): p. 137-47.

24.	Klos, K.L., et al., *Linkage analysis of plasma ApoE in three ethnic groups: multiple genes with context-dependent effects.* Ann Hum Genet, 2005. 69(Pt 2): p. 157-67.

25.	Sepehrnia, B., et al., *Genetic studies of human apolipoproteins. VIII. Role of the apolipoprotein H polymorphism in relation to serum lipoprotein concentrations.* Hum Genet, 1989. 82(2): p. 118-22.

26.	Lusis, A.J., R. Mar, and P. Pajukanta, *Genetics of atherosclerosis.* Annu Rev Genomics Hum Genet, 2004. 5: p. 189-218.

27.	Hibuse, T., et al., *Aquaporin 7 deficiency is associated with development of obesity through activation of adipose glycerol kinase.* Proc Natl Acad Sci U S A, 2005. 102(31): p. 10993-8.

28.	Aitman, T.J., et al., *Identification of Cd36 (Fat) as an insulin-resistance gene causing defective fatty acid and glucose metabolism in hypertensive rats.* Nat Genet, 1999. 21(1): p. 76-83.

29.	Wang, N.D., et al., *Impaired energy homeostasis in C/EBP alpha knockout mice.* Science, 1995. 269(5227): p. 1108-12.

30.	Boekholdt, S.M. and J.F. Thompson, *Natural genetic variation as a tool in understanding the role of CETP in lipid levels and disease.* J Lipid Res, 2003. 44(6): p. 1080-93.

31.	Turek, F.W., et al., *Obesity and metabolic syndrome in circadian Clock mutant mice*, in *Science*. 2005. p. 1043-5.

32.	Altomonte, J., et al., *Foxo1 mediates insulin action on apoC-III and triglyceride metabolism.* J Clin Invest, 2004. 114(10): p. 1493-503.

33.	Chasman, D.I., et al., *Pharmacogenetic study of statin therapy and cholesterol reduction.* Jama, 2004. 291(23): p. 2821-7.

34.	Istvan, E.S. and J. Deisenhofer, *Structural mechanism for statin inhibition of HMG-CoA reductase.* Science, 2001. 292(5519): p. 1160-4.

35.	Paterson, J.M., et al., *Metabolic syndrome without obesity: Hepatic overexpression of 11beta-hydroxysteroid dehydrogenase type 1 in transgenic mice.* Proc Natl Acad Sci U S A, 2004. 101(18): p. 7088-93.

36.	Takaishi, K., et al., *Hepatic insig-1 or -2 overexpression reduces lipogenesis in obese Zucker diabetic fatty rats and in fasted/refed normal rats.* Proc Natl Acad Sci U S A, 2004. 101(18): p. 7106-11.

37.	Cervino, A.C., et al., *Integrating QTL and high-density SNP analyses in mice to identify Insig2 as a susceptibility gene for plasma cholesterol levels.* Genomics, 2005. 86(5): p. 505-17.

38.	Funke, H., et al., *A molecular defect causing fish eye disease: an amino acid exchange in lecithin-cholesterol acyltransferase (LCAT) leads to the selective loss of alpha-LCAT activity.* Proc Natl Acad Sci U S A, 1991. 88(11): p. 4855-9.

39.	Knoblauch, H., et al., *Common haplotypes in five genes influence genetic variance of LDL and HDL cholesterol in the general population.* Hum Mol Genet, 2002. 11(12): p. 1477-85.

40.	Garcia, C.K., et al., *Autosomal recessive hypercholesterolemia caused by mutations in a putative LDL receptor adaptor protein.* Science, 2001. 292(5520): p. 1394-8.

41.	Sirinian, M.I., et al., *Adaptor protein ARH is recruited to the plasma membrane by low density lipoprotein (LDL) binding and modulates endocytosis of the LDL/LDL receptor complex in hepatocytes.* J Biol Chem, 2005. 280(46): p. 38416-23.

42.	Bouchard, C., et al., *Obesity Gene Map Database*. 2006.

43.	Wen, X.Y., et al., *Identification of a novel lipase gene mutated in lpd mice with hypertriglyceridemia and associated with dyslipidemia in humans.* Hum Mol Genet, 2003. 12(10): p. 1131-43.

44.	Speckman, R.A., et al., *Mutational and haplotype analyses of families with familial partial lipodystrophy (Dunnigan variety) reveal recurrent missense mutations in the globular C-terminal domain of lamin A/C.* Am J Hum Genet, 2000. 66(4): p. 1192-8.

45.	Lawn, R.M., et al., *Atherogenesis in transgenic mice expressing human apolipoprotein(a).* Nature, 1992. 360(6405): p. 670-2.

46.	Berg, K., G. Dahlen, and A.L. Borresen, *Lp(a) phenotypes, other lipoprotein parameters, and a family history of coronary heart disease in middle-aged males.* Clin Genet, 1979. 16(5): p. 347-52.

47.	Ichinose, A. and M. Kuriyama, *Detection of polymorphisms in the 5'-flanking region of the gene for apolipoprotein(a).* Biochem Biophys Res Commun, 1995. 209(1): p. 372-8.

48.	Ma, Y., et al., *A mutation in the human lipoprotein lipase gene as the most common cause of familial chylomicronemia in French Canadians.* N Engl J Med, 1991. 324(25): p. 1761-6.

49.	Wittrup, H.H., A. Tybjaerg-Hansen, and B.G. Nordestgaard, *Lipoprotein lipase mutations, plasma lipids and lipoproteins, and risk of ischemic heart disease. A meta-analysis.* Circulation, 1999. 99(22): p. 2901-7.

50.	Yang, W., et al., *Lipoprotein lipase gene is in linkage with blood pressure phenotypes in Chinese pedigrees.* Hum Genet, 2004. 115(1): p. 8-12.

51.	Peet, D.J., et al., *Cholesterol and bile acid metabolism are impaired in mice lacking the nuclear oxysterol receptor LXR alpha.* Cell, 1998. 93(5): p. 693-704.

52.	Raabe, M., et al., *Knockout of the abetalipoproteinemia gene in mice: reduced lipoprotein secretion in heterozygotes and embryonic lethality in homozygotes.* Proc Natl Acad Sci U S A, 1998. 95(15): p. 8686-91.

53.	Altmann, S.W., et al., *Niemann-Pick C1 Like 1 protein is critical for intestinal cholesterol absorption.* Science, 2004. 303(5661): p. 1201-4.

54.	Cohen, J.C., et al., *Multiple rare variants in NPC1L1 associated with reduced sterol absorption and plasma low-density lipoprotein levels.* Proc Natl Acad Sci U S A, 2006. 103(6): p. 1810-1815.

55.	Karvonen, M.K., et al., *Leucine7 to proline7 polymorphism in the preproneuropeptide Y is associated with the progression of carotid atherosclerosis, blood pressure and serum lipids in Finnish men.* Atherosclerosis, 2001. 159(1): p. 145-51.

56.	Urizar, N.L., et al., *A natural product that lowers cholesterol as an antagonist ligand for FXR.* Science, 2002. 296(5573): p. 1703-6.

57.	Abifadel, M., et al., *Mutations in PCSK9 cause autosomal dominant hypercholesterolemia.* Nat Genet, 2003. 34(2): p. 154-6.

58.	Maxwell, K.N. and J.L. Breslow, *Adenoviral-mediated expression of Pcsk9 in mice results in a low-density lipoprotein receptor knockout phenotype.* Proc Natl Acad Sci U S A, 2004. 101(18): p. 7100-5.

59.	Zimmermann, R., et al., *Fat mobilization in adipose tissue is promoted by adipose triglyceride lipase.* Science, 2004. 306(5700): p. 1383-6.

60.	Shih, D.M., et al., *Mice lacking serum paraoxonase are susceptible to organophosphate toxicity and atherosclerosis.* Nature, 1998. 394(6690): p. 284-7.

61.	Leus, F.R., et al., *PON2 gene variants are associated with clinical manifestations of cardiovascular disease in familial hypercholesterolemia patients.* Atherosclerosis, 2001. 154(3): p. 641-9.

62.	Sanghera, D.K., et al., *DNA polymorphisms in two paraoxonase genes (PON1 and PON2) are associated with the risk of coronary heart disease.* Am J Hum Genet, 1998. 62(1): p. 36-44.

63.	Pineda Torra, I., P. Gervois, and B. Staels, *Peroxisome proliferator-activated receptor alpha in metabolic disease, inflammation, atherosclerosis and aging.* Curr Opin Lipidol, 1999. 10(2): p. 151-9.

64.	Cheng, L., et al., *Cardiomyocyte-restricted peroxisome proliferator-activated receptor-delta deletion perturbs myocardial fatty acid oxidation and leads to cardiomyopathy.* Nat Med, 2004. 10(11): p. 1245-50.

65.	Hegele, R.A., et al., *PPARG F388L, a transactivation-deficient mutant, in familial partial lipodystrophy.* Diabetes, 2002. 51(12): p. 3586-90.

66.	Kuriyama, H., et al., *Compensatory increase in fatty acid synthesis in adipose tissue of mice with conditional deficiency of SCAP in liver.* Cell Metab, 2005. 1(1): p. 41-51.

67.	Osborne, T.F., *CREating a SCAP-less liver keeps SREBPs pinned in the ER membrane and prevents increased lipid synthesis in response to low cholesterol and high insulin.* Genes Dev, 2001. 15(15): p. 1873-8.

68.	Shimano, H., et al., *Elevated levels of SREBP-2 and cholesterol synthesis in livers of mice homozygous for a targeted disruption of the SREBP-1 gene.* J Clin Invest, 1997. 100(8): p. 2115-24.

69.	Osborne, T.F., *CREating a SCAP-less liver keeps SREBPs pinned in the ER membrane and prevents increased lipid synthesis in response to low cholesterol and high insulin*, in *Genes Dev*. 2001. p. 1873-8.

70.	Shimano, H., et al., *Elevated levels of SREBP-2 and cholesterol synthesis in livers of mice homozygous for a targeted disruption of the SREBP-1 gene*, in *J Clin Invest*. 1997. p. 2115-24.

71.	Babaya, N., et al., *Association of I27L polymorphism of hepatocyte nuclear factor-1 alpha gene with high-density lipoprotein cholesterol level.* J Clin Endocrinol Metab, 2003. 88(6): p. 2548-51.

72.	Geurts, J.M., et al., *Identification of TNFRSF1B as a novel modifier gene in familial combined hyperlipidemia.* Hum Mol Genet, 2000. 9(14): p. 2067-74.

73.	Coon, H., et al., *Upstream stimulatory factor 1 associated with familial combined hyperlipidemia, LDL cholesterol, and triglycerides.* Hum Genet, 2005. 117(5): p. 444-51.

74.	Pajukanta, P., et al., *Familial combined hyperlipidemia is associated with upstream transcription factor 1 (USF1).* Nat Genet, 2004. 36(4): p. 371-6.
