# Supplementary material for: Adaptations to Climate in Candidate Genes for Common Metabolic Disorders
Source: PLoS Genet. 2008 Feb 15;4(2):e32. doi: 10.1371/journal.pgen.0040032 (PMC2242814; doi:10.1371/journal.pgen.0040032)
Supplement: Supplementary file 5 [file pgen.0040032.st005.doc]

Supplementary Table 5. Literature review results for Metabolic Syndrome.

| Gene | Gene ID | Mendelian phenotype | Animal model | Common phenotype (associations) | | Linkage* | Physiology | Drug target |
| --- | --- | --- | --- | --- | --- | --- | --- | --- |
|  |  |  |  | Single analysis | Meta analysis |  |  |  |
| **ACE** | 1636 |  |  | [1] |  |  |  |  |
| **ADRB2** | 154 |  |  | [1] |  |  |  |  |
| **ALMS1** | 7840 | [2] | [3] |  |  |  |  |  |
| **APOC3** | 345 |  |  | [4] |  |  |  |  |
| **APOE** | 348 |  |  | [5] |  |  |  |  |
| **AQP7** | 364 |  | [6] |  |  |  |  |  |
| **CD36** | 948 |  | [7] |  |  |  |  |  |
| **CLOCK** | 9575 |  | [8] |  |  |  |  |  |
| **IL6** | 3569 |  |  | [1] |  |  |  |  |
| **FABP2** | 2169 |  |  | [4] |  |  |  |  |
| **SLC27A4** | 10999 |  |  | [9] |  |  |  |  |
| **FOXA2** | 3170 |  | [10] |  |  |  |  |  |
| **GHRL** | 51738 |  |  | [11] |  |  |  |  |
| **GJA4** | 2701 |  |  | [5] |  |  |  |  |
| **GNB3** | 2784 |  |  | [12] |  |  |  |  |
| **HSD11B1** | 3290 | [13] |  |  |  |  |  |  |
| **LMNA** | 4000 |  |  | [14] & [1] |  |  |  |  |
| **LTA** | 4049 |  |  | [1] |  |  |  |  |
| **MMP3** | 4314 |  |  | [5] |  |  |  |  |
| **SERPINE1** | 5054 |  |  | [15] |  |  |  |  |
| **PPARA** | 5465 |  |  | [1] |  |  |  |  |
| **PPARG** | 5468 | [16] |  | [14] & [1] |  |  | [17] |  |
| **ACSM3** | 6296 |  |  | [14] |  |  |  |  |
| **TNF** | 7124 |  |  | [18] & [19] |  |  |  |  |
| **TNFRSF1B** | 7133 |  |  | [14] |  |  |  |  |
| **UBL5** | 59286 |  |  | [14] |  |  |  |  |
| **USF1** | 7391 |  |  | [20] |  | [20] |  |  |

* Evidence was recorded for genes which lie under linkage peaks and are implicated by other types of evidence.

References:

1.	Meirhaeghe, A., et al., *Lack of association between certain candidate gene polymorphisms and the metabolic syndrome.* Mol Genet Metab, 2005. **86**(1-2): p. 293-9.

2.	Marshall, J.D., et al., *New Alstrom syndrome phenotypes based on the evaluation of 182 cases.* Arch Intern Med, 2005. **165**(6): p. 675-83.

3.	Collin, G.B., et al., *Alms1-disrupted mice recapitulate human Alstrom syndrome.* Hum Mol Genet, 2005. **14**(16): p. 2323-33.

4.	Guettier, J.M., et al., *Polymorphisms in the fatty acid-binding protein 2 and apolipoprotein C-III genes are associated with the metabolic syndrome and dyslipidemia in a South Indian population.* J Clin Endocrinol Metab, 2005. **90**(3): p. 1705-11.

5.	Hirashiki, A., et al., *Association of gene polymorphisms with coronary artery disease in low- or high-risk subjects defined by conventional risk factors.* J Am Coll Cardiol, 2003. **42**(8): p. 1429-37.

6.	Hibuse, T., et al., *Aquaporin 7 deficiency is associated with development of obesity through activation of adipose glycerol kinase.* Proc Natl Acad Sci U S A, 2005. **102**(31): p. 10993-8.

7.	Aitman, T.J., et al., *Identification of Cd36 (Fat) as an insulin-resistance gene causing defective fatty acid and glucose metabolism in hypertensive rats.* Nat Genet, 1999. **21**(1): p. 76-83.

8.	Turek, F.W., et al., *Obesity and metabolic syndrome in circadian Clock mutant mice.* Science, 2005. **308**(5724): p. 1043-5.

9.	Gertow, K., et al., *Genetic and structural evaluation of fatty acid transport protein-4 in relation to markers of the insulin resistance syndrome.* J Clin Endocrinol Metab, 2004. **89**(1): p. 392-9.

10.	Wolfrum, C., et al., *Foxa2 regulates lipid metabolism and ketogenesis in the liver during fasting and in diabetes.* Nature, 2004. **432**(7020): p. 1027-32.

11.	Steinle, N.I., et al., *Variants in the ghrelin gene are associated with metabolic syndrome in the Old Order Amish.* J Clin Endocrinol Metab, 2005. **90**(12): p. 6672-7.

12.	Brand, E., et al., *An epidemiological study of blood pressure and metabolic phenotypes in relation to the Gbeta3 C825T polymorphism.* J Hypertens, 2003. **21**(4): p. 729-37.

13.	Masuzaki, H., et al., *A transgenic model of visceral obesity and the metabolic syndrome.* Science, 2001. **294**(5549): p. 2166-70.

14.	Bouchard, C., et al., *Obesity Gene Map Database*. 2006.

15.	Lopes, C., et al., *PAI-1 polymorphisms modulate phenotypes associated with the metabolic syndrome in obese and diabetic Caucasian population.* Diabetologia, 2003. **46**(9): p. 1284-90.

16.	Barroso, I., et al., *Dominant negative mutations in human PPARgamma associated with severe insulin resistance, diabetes mellitus and hypertension.* Nature, 1999. **402**(6764): p. 880-3.

17.	Schupp, M., et al., *Angiotensin type 1 receptor blockers induce peroxisome proliferator-activated receptor-gamma activity*, in *Circulation*. 2004. p. 2054-7.

18.	Dalziel, B., et al., *Association of the TNF-alpha -308 G/A promoter polymorphism with insulin resistance in obesity.* Obes Res, 2002. **10**(5): p. 401-7.

19.	Wybranska, I., et al., *The TNF-alpha gene NcoI polymorphism at position -308 of the promoter influences insulin resistance, and increases serum triglycerides after postprandial lipaemia in familiar obesity.* Clin Chem Lab Med, 2003. **41**(4): p. 501-10.

20.	Ng, M.C., et al., *The linkage and association of the gene encoding upstream stimulatory factor 1 with type 2 diabetes and metabolic syndrome in the Chinese population.* Diabetologia, 2005. **48**(10): p. 2018-24.
