# Supplementary material for: Adaptations to Climate in Candidate Genes for Common Metabolic Disorders
Source: PLoS Genet. 2008 Feb 15;4(2):e32. doi: 10.1371/journal.pgen.0040032 (PMC2242814; doi:10.1371/journal.pgen.0040032)
Supplement: Supplementary file 6 [file pgen.0040032.st006.doc]

**Selection of candidate genes for genotyping**

Table S6. Genes selected from the network analysis in which tag SNPs were genotyped

| GeneID | GeneSymbol | GeneName |  |
| --- | --- | --- | --- |
| 118 | *ADD1* | adducin 1 (alpha) | seed |
| 151 | *ADRA2B* | adrenergic, alpha-2B-, receptor | seed |
| 185 | *AGTR1* | angiotensin II receptor, type 1 | seed |
| 338 | *APOB* | apolipoprotein B (including Ag(x) antigen) | seed |
| 948 | *CD36* | CD36 antigen (collagen type I receptor, thrombospondin receptor) | seed |
| 1071 | *CETP* | cholesteryl ester transfer protein, plasma | seed |
| 1636 | *ACE* | angiotensin I converting enzyme (peptidyl-dipeptidase A) 1 | seed |
| 2053 | *EPHX2* | epoxide hydrolase 2, cytoplasmic | seed |
| 2169 | *FABP2* | fatty acid binding protein 2, intestinal | seed |
| 2778 | *GNAS* | GNAS complex locus | seed |
| 3290 | *HSD11B1* | hydroxysteroid (11-beta) dehydrogenase 1 | seed |
| 3375 | *IAPP* | islet amyloid polypeptide | seed |
| 3569 | *IL6* | interleukin 6 (interferon, beta 2) | seed |
| 3651 | *IPF1* | insulin promoter factor 1, homeodomain transcription factor | seed |
| 3667 | *IRS1* | insulin receptor substrate 1 | seed |
| 3758 | *KCNJ1* | potassium inwardly-rectifying channel, subfamily J, member 1 | seed |
| 3767 | *KCNJ11* | potassium inwardly-rectifying channel, subfamily J, member 11 | seed |
| 3931 | *LCAT* | lecithin-cholesterol acyltransferase | seed |
| 3949 | *LDLR* | low density lipoprotein receptor (familial hypercholesterolemia) | seed |
| 3991 | *LIPE* | lipase, hormone-sensitive | seed |
| 4018 | *LPA* | lipoprotein, Lp(a) | seed |
| 4023 | *LPL* | lipoprotein lipase | seed |
| 4049 | *LTA* | lymphotoxin alpha (TNF superfamily, member 1) | seed |
| 4843 | *NOS2A* | nitric oxide synthase 2A (inducible, hepatocytes) | seed |
| 4878 | *NPPA* | natriuretic peptide precursor A | seed |
| 5080 | *PAX6* | paired box gene 6 (aniridia, keratitis) | seed |
| 5122 | *PCSK1* | proprotein convertase subtilisin/kexin type 1 | seed |
| 5444 | *PON1* | paraoxonase 1 | seed |
| 5465 | *PPARA* | peroxisome proliferative activated receptor, alpha | seed |
| 5467 | *PPARD* | peroxisome proliferative activated receptor, delta | seed |
| 5770 | *PTPN1* | protein tyrosine phosphatase, non-receptor type 1 | seed |
| 6296 | *ACSM3* | acyl-CoA synthetase medium-chain family member 3 | seed |
| 6833 | *ABCC8* | ATP-binding cassette, sub-family C (CFTR/MRP), member 8 | seed |
| 6927 | *TCF1* | transcription factor 1, hepatic; LF-B1, hepatic nuclear factor (HNF1), albumin proximal factor | seed |
| 7133 | *TNFRSF1B* | tumor necrosis factor receptor superfamily, member 1B | seed |
| 7350 | *UCP1* | uncoupling protein 1 (mitochondrial, proton carrier) | seed |
| 7352 | *UCP3* | uncoupling protein 3 (mitochondrial, proton carrier) | seed |
| 9388 | *LIPG* | lipase, endothelial | seed |
| 9971 | *NR1H4* | nuclear receptor subfamily 1, group H, member 4 | seed |
| 12 | *SERPINA3* | serpin peptidase inhibitor, clade A (alpha-1 antiproteinase, antitrypsin), member 3 | non-seed |
| 267 | *AMFR* | autocrine motility factor receptor | non-seed |
| 590 | *BCHE* | butyrylcholinesterase | non-seed |
| 950 | *SCARB2* | scavenger receptor class B, member 2 | non-seed |
| 1357 | *CPA1* | carboxypeptidase A1 (pancreatic) | non-seed |
| 1385 | *CREB1* | cAMP responsive element binding protein 1 | non-seed |
| 1432 | *MAPK14* | mitogen-activated protein kinase 14 | non-seed |
| 1813 | *DRD2* | dopamine receptor D2 | non-seed |
| 1827 | *DSCR1* | Down syndrome critical region gene 1 | non-seed |
| 1956 | *EGFR* | epidermal growth factor receptor (erythroblastic leukemia viral (v-erb-b) oncogene homolog, avian) | non-seed |
| 2052 | *EPHX1* | epoxide hydrolase 1, microsomal (xenobiotic) | non-seed |
| 2185 | *PTK2B* | PTK2B protein tyrosine kinase 2 beta | non-seed |
| 2247 | *FGF2* | fibroblast growth factor 2 (basic) | non-seed |
| 2952 | *GSTT1* | glutathione S-transferase theta 1 | non-seed |
| 3552 | *IL1A* | interleukin 1, alpha | non-seed |
| 3576 | *IL8* | interleukin 8 | non-seed |
| 4205 | *MEF2A* | MADS box transcription enhancer factor 2, polypeptide A (myocyte enhancer factor 2A) | non-seed |
| 5290 | *PIK3CA* | phosphoinositide-3-kinase, catalytic, alpha polypeptide | non-seed |
| 5291 | *PIK3CB* | phosphoinositide-3-kinase, catalytic, beta polypeptide | non-seed |
| 5294 | *PIK3CG* | phosphoinositide-3-kinase, catalytic, gamma polypeptide | non-seed |
| 5319 | *PLA2G1B* | phospholipase A2, group IB (pancreas) | non-seed |
| 5320 | *PLA2G2A* | phospholipase A2, group IIA (platelets, synovial fluid) | non-seed |
| 5594 | *MAPK1* | mitogen-activated protein kinase 1 | non-seed |
| 5599 | *MAPK8* | mitogen-activated protein kinase 8 | non-seed |
| 5664 | *PSEN2* | presenilin 2 (Alzheimer disease 4) | non-seed |
| 6646 | *SOAT1* | sterol O-acyltransferase (acyl-Coenzyme A: cholesterol acyltransferase) 1 | non-seed |
| 6647 | *SOD1* | superoxide dismutase 1, soluble (amyotrophic lateral sclerosis 1 (adult)) | non-seed |
| 6667 | *SP1* | Sp1 transcription factor | non-seed |
| 7031 | *TFF1* | trefoil factor 1 (breast cancer, estrogen-inducible sequence expressed in) | non-seed |
| 9159 | *PCSK7* | proprotein convertase subtilisin/kexin type 7 | non-seed |
| 10498 | *CARM1* | coactivator-associated arginine methyltransferase 1 | non-seed |
| 10598 | *AHSA1* | AHA1, activator of heat shock 90kDa protein ATPase homolog 1 (yeast) | non-seed |
| 11162 | *NUDT6* | nudix (nucleoside diphosphate linked moiety X)-type motif 6 | non-seed |
| 22915 | *MMRN1* | multimerin 1 | non-seed |
| 115677 | *NOSTRIN* | nitric oxide synthase trafficker | non-seed |
