# Supplementary material for: Adaptations to Climate in Candidate Genes for Common Metabolic Disorders
Source: PLoS Genet. 2008 Feb 15;4(2):e32. doi: 10.1371/journal.pgen.0040032 (PMC2242814; doi:10.1371/journal.pgen.0040032)
Supplement: Supplementary file 7 [file pgen.0040032.st007.doc]

**Eigenvectors from Principal Components Analysis**

Table S7a. Eigenvectors for the first two principle components for PCA with summer variables.

| **variable** | **Summer PC1** | **Summer PC2** |
| --- | --- | --- |
| Absolute Latitude | 0.360 | -0.228 |
| Longitude | -0.247 | -0.057 |
| Summer Minimum T | -0.326 | 0.436 |
| Summer Maximum T | 0.021 | 0.568 |
| Summer Mean T | -0.102 | 0.561 |
| Summer Precipitation Rate | -0.490 | -0.144 |
| Summer SWRF | 0.514 | 0.117 |
| Summer Relative Humidity | -0.434 | -0.288 |

Table S7b. Eigenvectors for the first two principle components of PCA with winter variables.

| **variable** | **Winter PC1** | **Winter PC2** |
| --- | --- | --- |
| Absolute Latitude | 0.434 | -0.031 |
| Longitude | -0.067 | 0.064 |
| Winter Minimum T | -0.434 | 0.148 |
| Winter Maximum T | -0.452 | -0.071 |
| Winter Mean T | -0.445 | 0.057 |
| Winter Precipitation Rate | -0.197 | 0.643 |
| Winter SWRF | -0.397 | -0.252 |
| Winter Relative Humidity | 0.146 | 0.698 |
