# Supplementary material for: Adaptations to Climate in Candidate Genes for Common Metabolic Disorders
Source: PLoS Genet. 2008 Feb 15;4(2):e32. doi: 10.1371/journal.pgen.0040032 (PMC2242814; doi:10.1371/journal.pgen.0040032)
Supplement: Supplementary file 8 [file pgen.0040032.st008.doc]

**Proportion of variance explained by each principal component**

Table S8a. Proportion of variance explained by each of the summer principal components. For analyses, we used the first two summer principal components, which explain 77.6% of the variance in the matrix of climate variables.

|  | PC1 | PC2 | PC3 | PC4 | PC5 | PC6 | PC7 | PC8 |
| --- | --- | --- | --- | --- | --- | --- | --- | --- |
| Standard deviation | 1.791 | 1.731 | 0.937 | 0.707 | 0.484 | 0.370 | 0.202 | 0.081 |
| Proportion of Variance | 0.401 | 0.375 | 0.110 | 0.063 | 0.029 | 0.017 | 0.005 | 0.001 |
| Cumulative Proportion | 0.401 | 0.776 | 0.885 | 0.948 | 0.977 | 0.994 | 0.999 | 1.000 |

Table S8b. Proportion of variance explained by each of the winter principal components. For analyses, we used the first two summer principal components, which explain 78.6% of the variance in the matrix of climate variables.

|  | PC1 | PC2 | PC3 | PC4 | PC5 | PC6 | PC7 | PC8 |
| --- | --- | --- | --- | --- | --- | --- | --- | --- |
| Standard deviation | 2.163 | 1.268 | 0.996 | 0.670 | 0.486 | 0.172 | 0.076 | 0.033 |
| Proportion of Variance | 0.585 | 0.201 | 0.124 | 0.056 | 0.030 | 0.004 | 0.001 | 0.000 |
| Cumulative Proportion | 0.585 | 0.786 | 0.910 | 0.966 | 0.995 | 0.999 | 1.000 | 1.000 |
