# Supplementary material for: Adaptations to Climate in Candidate Genes for Common Metabolic Disorders
Source: PLoS Genet. 2008 Feb 15;4(2):e32. doi: 10.1371/journal.pgen.0040032 (PMC2242814; doi:10.1371/journal.pgen.0040032)
Supplement: Supplementary file 9 [file pgen.0040032.st009.doc]

**Proportion of SNPs significant with *RAPTOR* included in the analysis**

Table S9. Proportion of SNPs significant at different type I error levels. Values are bold if there is an excess in at least 95% of the bootstrap replicates when bootstrapping was performed across genes.

|  |  | α = | | | |
| --- | --- | --- | --- | --- | --- |
| method and variable | | 0.01 | 0.05 | 0.1 | 0.5 |
| Bayesian spatial analysis | |  |  |  |  |
|  | Absolute Latitude | 0.0250 | 0.0852 | 0.1500 | **0.6193** |
|  | Summer PC1 | **0.0523** | **0.1023** | **0.1580** | 0.5182 |
|  | Summer PC2 | 0.0205 | 0.0545 | 0.1057 | 0.4727 |
|  | Winter PC1 | 0.0432 | **0.1261** | **0.1807** | **0.6102** |
|  | Winter PC2 | 0.0125 | 0.0352 | 0.0977 | 0.4943 |
|  | Subsistence | 0.0170 | **0.1102** | **0.1557** | **0.6023** |
| Spearman rank correlation: | |  |  |  |  |
|  | Absolute Latitude | 0.0375 | **0.0852** | 0.1330 | 0.5500 |
|  | Summer PC1 | 0.0193 | 0.0568 | 0.1227 | 0.4773 |
|  | Summer PC2 | 0.0193 | 0.0693 | 0.1193 | 0.5784 |
|  | Winter PC1 | **0.0352** | 0.0898 | **0.1693** | **0.5966** |
|  | Winter PC2 | **0.0295** | 0.0432 | 0.0705 | 0.5261 |
